# Unusual derivatives from *Hypericum scabrum*

**DOI:** 10.1038/s41598-020-79305-y

**Published:** 2021-01-14

**Authors:** Sara Soroury, Mostafa Alilou, Thomas Gelbrich, Marzieh Tabefam, Ombeline Danton, Samad N. Ebrahimi, Marcel Kaiser, Matthias Hamburger, Hermann Stuppner, Mahdi Moridi Farimani

**Affiliations:** 1 Department of Phytochemistry, Faculty of Science, Golestan University, 15759-49138 Gorgan, Iran; 2 Department of Phytochemistry, Medicinal Plants and Drugs Research Institute, Shahid Beheshti University, Evin, Tehran, Iran; 3 Institute of Pharmacy/Pharmacognosy, Center for Molecular Biosciences Innsbruck, University of Innsbruck, Innrain 80/82, 6020 Innsbruck, Austria; 4 Institute of Pharmacy, Pharmaceutical Technology, University of Innsbruck, Innrain 52c, 6020 Innsbruck, Austria; 5 Division of Pharmaceutical Biology, University of Basel, Klingelbergstrasse 50, 4056 Basel, Switzerland; 6 Swiss Tropical and Public Health Institute, Socinstrasse 57, 4002 Basel, Switzerland

**Keywords:** Structure elucidation, Natural products

## Abstract

Three new compounds (**1**–**3**) with unusual skeletons were isolated from the *n*-hexane extract of the air-dried aerial parts of *Hypericum scabrum*. Compound **1** represents the first example of an esterified polycyclic polyprenylated acylphloroglucinol that features a unique tricyclo-[4.3.1.1^1,4^]-undecane skeleton. Compound **2** is a fairly simple MPAP, but with an unexpected cycloheptane ring decorated with prenyl substituents, and compound **3** has an unusual 5,5-spiroketal lactone core. Their structures were determined by extensive spectroscopic and spectrometric techniques (1D and 2D NMR, HRESI-TOFMS). Absolute configurations were established by ECD calculations, and the absolute structure of **2** was confirmed by a single crystal determination. Plausible biogenetic pathways of compounds **1**–**3** were also proposed. The in vitro antiprotozoal activity of the compounds against *Trypanosoma brucei rhodesiense* and *Plasmodium falciparum* and cytotoxicity against rat myoblast (L6) cells were determined. Compound **1** showed a moderate activity against *T. brucei* and *P. falciparum*, with IC_50_ values of 3.07 and 2.25 μM, respectively.

## Introduction

*Hypericum perforatum* has gained great attention in the scientific community due to its high economic value, and prompted detailed phytochemical investigation into other *Hypericum* species^1,2^. The *Hypericum* genus belonging to the Hypericaceae family with a wide distribution in temperate regions has been utilized in folk medicine of different parts of the globe^1,3,4^. These plants are known to include several types of compounds such as prenylated acylphloroglucinols, terpenoids, flavonoids, xanthones, naphtodianthrones^5,6^, and some spiro compounds including spiroterpenoids^7^, spirolactones^8^, polyprenylated spirocyclic acylphloroglucinols^9^, and spiroketals^10^. These metabolites are also reported to display a wide range of pharmacological properties including antimicrobial, antitumor, antioxidant, anti-HIV, and antidepressant activities^11–13^.

**Figure 1:**
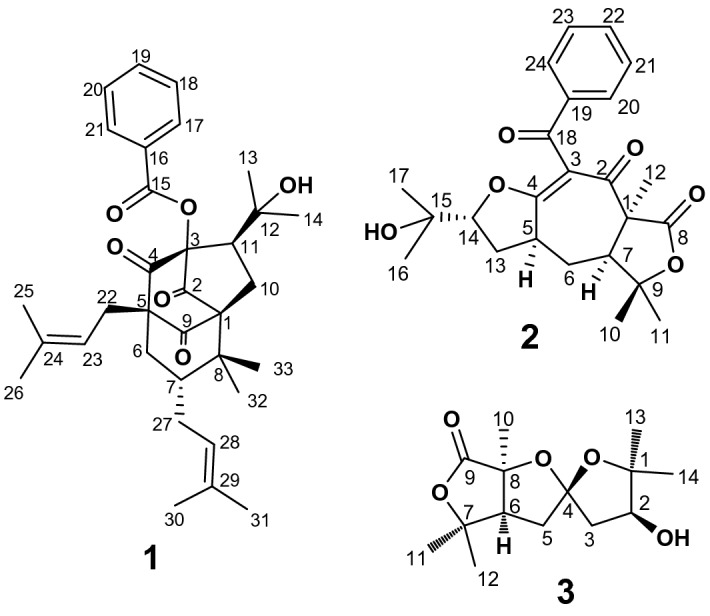
Structures of compounds **1**–**3**.

*Hypericum scabrum* has been used in Iranian folk medicine as an antiseptic, analgesic, sedative, and for treating headaches^14^. Prior phytochemical and pharmacological investigations into this species have demonstrated that the plant is rich in secondary metabolites, especially polycyclic polyprenylated acylphloroglucinols (PPAPs)^15–18^. PPAPs are a class of synthetically challenging and structurally attractive natural compounds having an acylphloroglucinol-derived core modified with prenyl substituents, which display diverse pharmacological activities^19^. In course of a systematic exploration for new bioactive secondary metabolites with novel structure^20–22^, three unprecedented structures (**1**–**3**, Fig. 1) were isolated from the aerial parts of *H. scabrum*. Although there are many examples of homo-adamantane PPAPs with a tricyclo[4.3.1.1^5,7^]-undecane skeleton^12,23,24^, compound **1** illustrates the first example of an esterified PPAP that features an unrivaled tricyclo-[4.3.1.1^1,4^]-undecane skeleton. Compound **2** is a reasonably simple monocyclic polyprenylated acylphloroglucinol (MPAP) with a unique cycloheptane ring decorated with prenyl substituents, and compound **3** has an unusual 5,5-spiroketal lactone core.

We here report on the structure elucidation of these compounds, including their absolute configuration, proposed biosynthetic pathways, and on the evaluation of their biological activity.

## Results and discussion

**Figure 2:**
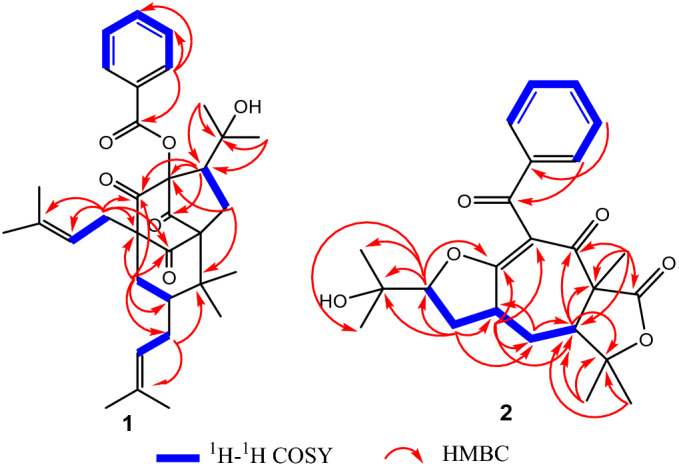
^1^H–^1^H COSY and key HMBC correlations of **1** and **2**.

Compound **1** was obtained as yellow gum {[α] ^25^_D_ + 17.2 (*c* = 1.0, CH_3_OH)}. Its molecular formula was deduced as C_33_H_42_O_6_ by HR-ESIMS at *m/z* 535.3067 [M + H]^+^ (calcd 535.3054), indicating 13 degrees of unsaturation. The ^1^H NMR spectrum of **1** (Table 1) revealed signals of eight methyl singlets (*δ*_H_ 1.00, 1.05, 1.37, 1.46, 1.50 , 1.54, 1.58, 1.65), two olefinic protons (*δ*_H_ 5.26, 1H, t, *J* = 7.4 Hz; 4.91, 1H, m), and a phenyl group (*δ*_H_ 7.87, 2H, dd, *J* = 8.4, 1.2 Hz; 7.51, 1H, t, *J* = 7.5 Hz; 7.36, 2H, t, *J* = 7.8 Hz). Analysis of the ^13^C NMR (Table 1) and HSQC spectra of **1** exhibited 33 carbon signals comprising three carbonyl groups [*δ*_C_ 203.3, 201.4, 200.9], a benzoate group [*δ*_C_ 165.6, 134.0, 129.9 × 2, 128.7 × 2, 127.9], eight methyls, four methylenes, four methines, and six quaternary carbons. The aforementioned data indicated some structural aspects of 7-*epi*-clusianone (**4**, Fig. 5), a BPAP with a normal bicyclo[3.3.1]-nonane core^25^, that was also isolated in this study^26^. The main spectral differences between **1** and **4** were due to the significant variations in the NMR data of the perenyl group at C-1, replacement of a benzylic ketone at *δ*_C_ 197.7 with a benzylic ester at *δ*_C_ 165.6, and replacement of an enolic carbon at *δ*_C_ 196.5 with a ketonic one at *δ*_C_ 200.9. Detailed analysis of the ^1^H and ^13^C NMR spectra indicated that a [CHC(CH_3_)_2_OH] group in **1** replaced the isobutenyl moiety at C_10_ in **4**. This deduction was corroborated by the key HMBC correlations from H_3_-13 (*δ*_H_ 1.37) and H_3_-14 (*δ*_H_ 1.46) to C-12 (*δ*_C_ 75.0) and C-11 (*δ*_C_ 45.4), coupled with the ^1^H-^1^H COSY correlation between H-11 (*δ*_H_ 2.11) and H_2_-10 (*δ*_H_ 1.92 and 1.98), as shown in Fig. 2. Additionally, HMBC correlations from H-11 to C-3 (*δ*_C_ 89.5), C-4 (*δ*_C_ 200.9), C-2 (*δ*_C_ 201.4), and from H_2_-10 to C-3 and C-8 (*δ*_C_ 46.3), demonstrated the foundation of a five-membered ring by the connection of C-11 to C-3. In the HMBC spectrum, correlations from aromatic protons (*δ*_H_ 7.87) to the resonance of a carbonyl group at (*δ*_C_ 165.6), supported by the upfield shift of C-3 resonance (*δ*_C_ 89.5) compared to it at **4** (*δ*_C_ 115.9), suggested the displacement of a benzoate ester at C-3 instead of the phenyl ketone. Moreover, HMBC correlations from H_2_-22 and H_2_-6 to the carbonyl resonances at *δ*_C_ 200.9 and *δ*_C_ 203.3 confirmed the position of the two other ketone groups at C-4 and C-9. The remaining parts of the molecule were similar to those of **4**. Thus, the gross structure of **1** was established as depicted in Fig. 1.

**Table 1:** ^1^H and ^13^C NMR data (CDCl_3_) of **1** (^1^H (500 MHz) and ^13^C (125 MHz)) and **2** (^1^H (600 MHz) and ^13^C (150 MHz).) (*δ* in ppm, *J* in Hz).

No.	**1**		No.	**2**	
	*δ*_C_	*δ*_H_, mult		*δ*_C_	*δ*_H_, mult
1	72.6		1	62.4	
2	201.4		2	193.7	
3	89.5		3	113.5	
4	200.9		4	179.0	
5	64.9		5	40.8	3.61 m
6*α*	36.1	1.85 d (14.6)	6*α*	31.6	2.13 ddd (13.7, 6.9, 1.7)
6*β*		2.45 dd (14.6, 6.5)	6*β*		1.83 m
7	41.8	2.05 t (7.4)	7	55.0	2.43 dd (12.1, 6.9)
8	46.3		8	174.1	
9	203.3		9	83.6	
10*α*	26.9	1.98^a^	10	23.6	1.20 s
10*β*		1.92^a^			
11	45.4	2.11 dd (10.7, 8.5)	11	28.5	1.39 s
12	75.0		12	26.1	1.77 s
13	27.7	1.37 s	13*α*	32.1	2.52 ddd (12.9, 9.0, 1.2)
			13*β*		1.90 ddd (12.9, 11.0, 9.2)
14	31.2	1.46 s	14	90.1	4.25 dd (9.2, 1.2)
15	165.6		15	72.6	
16	127.9		16	26.3	1.12 s
17,21	129.9	7.87 dd (8.4, 1.2)	17	26.4	1.05 s
18,20	128.7	7.36 t (7.8)	18	193.5	
19	134.0	7.51 t (7.5)	19	137.3	
22	28.7	2.53^c^	20,24	128.9	7.77 dd (8.3, 1.2)
23	119.3	5.26 t (7.4)	21,23	128.5	7.35 t (7.7)
24	134.8		22	133.2	7.46 t (7.4)
25	26.0	1.65 s			
26	18.0	1.58 s			
27a	25.5	2.42 dd (12.9, 5.2)			
27b		2.53^a^			
28	120.0	4.91 tq (5.7, 1.2)			
29	131.9				
30	25.9	1.50 s			
31	17.8	1.54 s			
32	25.3	1.00 s			
33	23.4	1.05 s			

^a^Overlapping signals.

**Figure 3:**
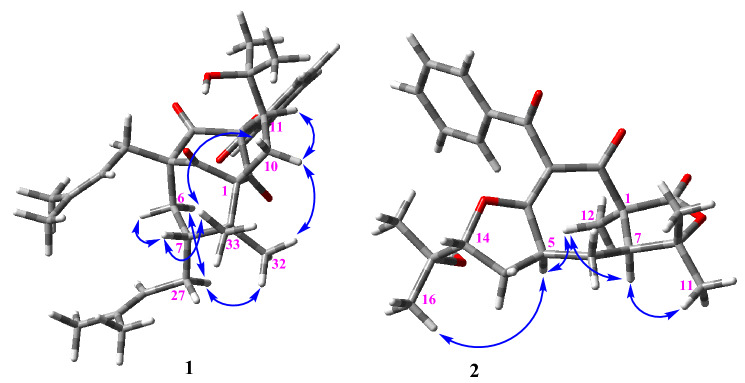
NOESY correlations of **1** and **2**.

**Figure 4:**
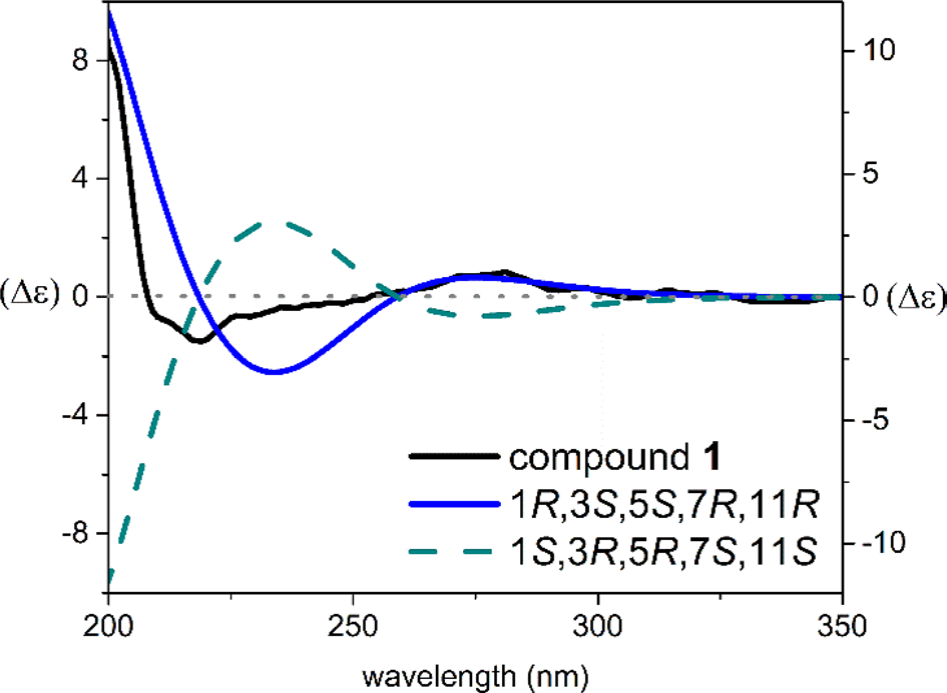
Comparison of experimental and TDDFT calculated ECD spectra of **1**.

The NOESY spectrum (Fig. 3) demonstrated correlations from H-7 to H-6*β* and H_3_-33, and from H_3_-33 to H-10*β* and certified their cofacial orientation. Also, diagnostic cross-peaks from H-27a to H_3_-32 and H-6*α*, from H_3_-32 to H-10*α*, and from H-10*α* to H-11, revealed their equal orientation. The absolute stereochemistry was specified by comparing the calculated ECD spectra with the experimental one. The experimental data showed two positive Cotton effects (CE) at 280 and 205 nm, along with negative CE at 220 nm (Fig. 4). The calculated ECD spectrum for 1*R*, 3*S*, 5*S*, 7*R*, 11*R* showed a good compatibility with the experimental data.

**Figure 5:**
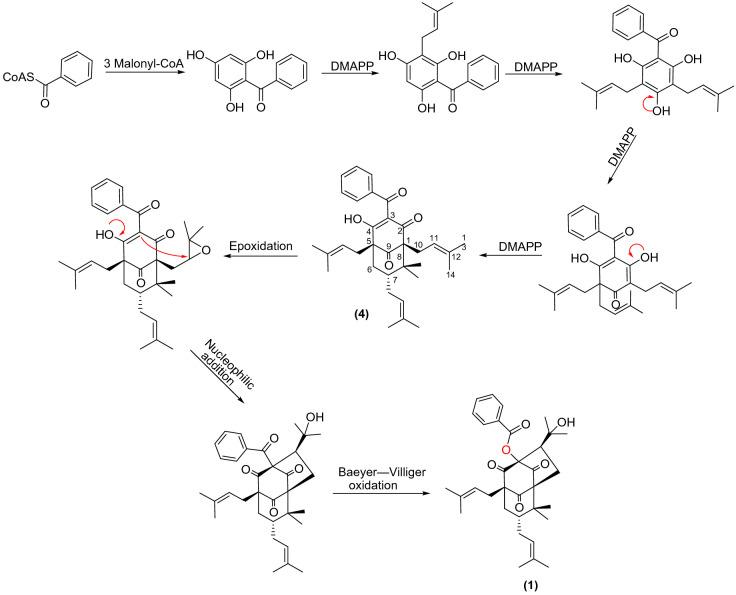
Putative biosynthetic pathway to **1**.

The hypothetical biogenetic pathway of **1** is proposed in Fig. 5. 7-*epi*-clusianone (**4**), an *endo*-bicyclic polyprenylated acylphloroglucinol (*endo*-BPAP) is assumed to be a precursor. The structural novelty of **1** involves ring closure of the enone moiety upon the epoxy function of the prenyl chain at C-1, to form a rigid caged tetracyclo-[4.3.1.1^1,4^]-undecane skeleton. Furthermore, a Baeyer–Villiger oxidation is required to create **1** as the first esterified PPAP in nature.

Compound **2** was obtained as a white powder {[α] ^25^_D_ + 30.5 (*c* = 1.0, CHCl_3_)}. Its molecular formula was distinguished as C_24_H_28_O_6_ by HR-ESIMS at *m/z* 413.1941 [M + H]^+^ (calcd 413.1959), proposing 11 degrees of unsaturation. The ^1^H NMR spectrum of **2** (Table 1) displayed signals of a mono-substituted phenyl group (*δ*_H_ 7.77, 2H, dd, *J* = 8.3, 1.2 Hz; 7.46, 1H, t, *J* = 7.4 Hz; 7.35, 2H, t, *J* = 7.7 Hz), five methyl singlets (*δ*_H_ 1.05, 1.12, 1.20, 1.39, 1.77), two diastereotopic methylenes [(*δ*_H_ 1.83, 1H, m; 2.13, 1H, ddd, *J* = 13.7, 6.9, 1.7 Hz); and (*δ*_H_ 1.90, 1H, ddd, *J* = 12.9, 11.0, 9.2 Hz; 2.52, 1H, ddd, *J* = 12.9, 9.0, 1.2 Hz)], and three methines including one oxygenated (*δ*_H_ 2.43, 1H, dd, *J* = 12.1, 6.9 Hz; 3.61, 1H, m; 4.25, 1H, dd, *J* = 9.2, 1.2 Hz). The ^13^C spectrum of **2** (Table 1) showed 24 carbon signals including five methyls, two methylenes, eight methines (five aromatics) and nine quaternary carbons (one aromatic). Accordingly, 27 protons could be numerated, while the unaccounted one could be due to the presence of an OH substituent in the structure. Deshielded ^13^C NMR resonances at *δ*_C_ 193.7 (C-2), 113.5 (C-3), and 179.0 (C-4) proposed the attendance of an *α*,*β*-unsaturated ketone moiety comprising a tetrasubstituted olefinic group and an oxygen substituent at the *β*-position. The resonances at *δ*_C_ 174.1 (C-8) and 193.5 (C-18) were indicative of two other carbonyl groups. Three carbon signals at *δ*_C_ 72.6 (C-15), 83.6 (C-9), and 90.1 (C-14) indicated the entity of oxygenbearing *sp*^3^ carbons. Considering the number of *sp*^2^ or *sp* carbon resonances and 11 degrees of hydrogen deficiency, a tricyclic core structure for compound **2** was clearly deduced that possesses a seven-membered carbocyclic ring, a cyclic ether and a lactone moiety. By interpreting COSY correlations (Fig. 2), it was thinkable to create a long proton connection from H-7 to H-14 through H_2_-6, H-5, and H_2_-13. The HMBC spectrum showed correlations from H-5 to C-3, C-4, C-6, and C-7, and from H-7 to C-1 and C-2, proving the seven-member carbocyclic ring as the core of the structure. HMBC correlations from H-14 to C-4, C-15, C-16, and C-17 resulted in the construction of the ether ring with a substituted 2-propanol moiety. HMBC cross-peaks from H_3_-10 and H_3_-11 to C-9 and C-7, and from H-7 to C-9, corroborated the nature of the lactone moiety. The fifth methyl group was placed at C-1 pursuant to the HMBC cross-peaks of H_3_-12 with C-1, C-2, C-7, and C-8. In the HMBC spectrum, correlations from aromatic protons (*δ*_H_ 7.77) to the resonance of a carbonyl group at (*δ*_C_ 193.5) supported the presence of the benzoyl moiety at C-3. However, no direct HMBC correlations to C-3 were observed.

**Figure 6:**
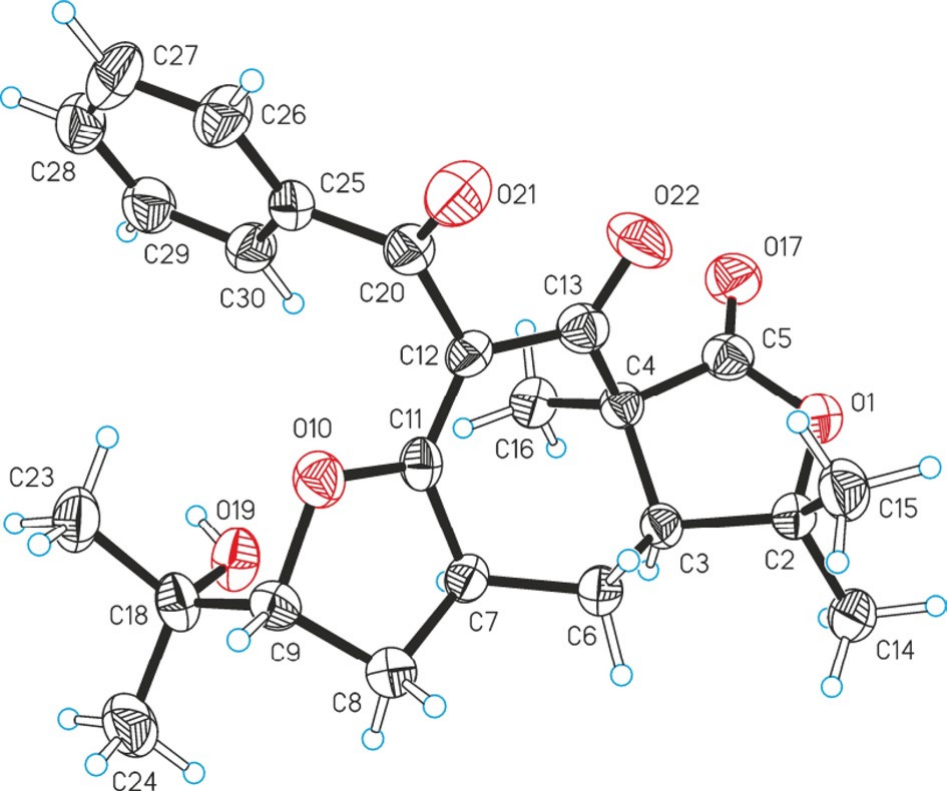
Molecular structure of **2** (non-H atoms are represented as thermal ellipsoids drawn at the 50% level).

The relative stereochemistry of **2** was resolved by inspecting the NOESY spectrum (Fig. 3). In the NOESY spectrum, H_3_-12 showed NOE correlations with H-7 and H-5, indicating their *α*-orientations. The fact that no NOE correlation peaks were found between H-5 and H-14, and instead, a cross-peak was detected between H-5 and H_3_-16, indicated the *β* orientation of H-14. Conclusive evidence for the postulated structure of **2** was obtained from a single-crystal X-ray structure analysis, which precisely confirmed the absolute configuration as (1*S*, 5*S*, 7*S*, 14*R*) (Fig. 6).

Many bi- and tricyclic PPAPs have been reported to occur in nature, however compound **2** is the first monocyclic polyprenylated acylphloroglucinol (MPAP) with a cycloheptane ring decorated with prenyl substituents.

**Figure 7:**
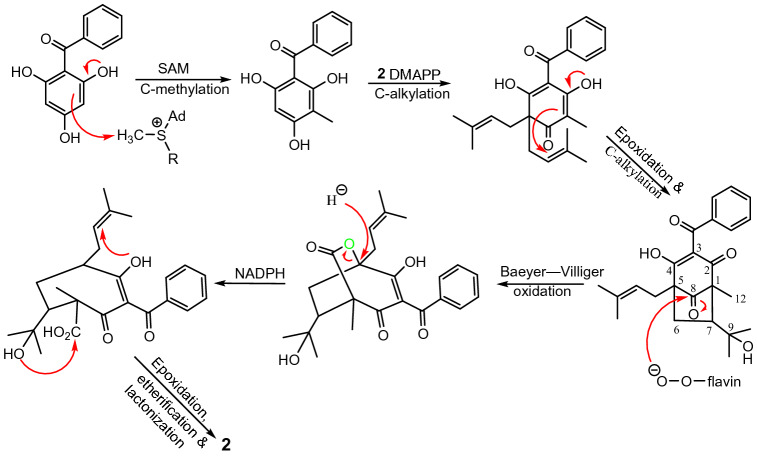
Putative biosynthesic pathway to **2**.

Biosynthetically, compound **2** is presumably derived from a common phloroglucinol core (Fig. 7) via methylation at C-1 and diprenylation at C-5, followed by the C-alkylation of the epoxide intermediate to form the C_1_–C_7_ bond. This intermediate contains a bicyclo[3.2.1]octane-2,4,8-trione core that was found in a smaller group of PPAPs^27^. A Baeyer–Villiger oxidation, followed by a ring opening through the nucleophilic attachment of NADPH, would then form the cycloheptane key ring. Finally, successive formation of the hydrofuran and lactone rings via etherification and lactonization of the side chains would lead to compound **2**.

**Figure 8:**
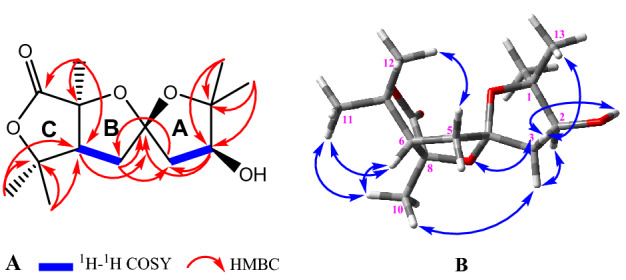
(**A**) ^1^H–^1^H COSY and key HMBC and (**B**) NOESY correlations of **3**.

Compound **3**, colorless oil {[α] ^25^_D_ + 9.1 (c = 1.0, CH_3_OH)}, showed an accurate HR-ESIMS ion at *m/z* 293.1366 [M + Na]^+^ (calcd 293.1365), revealing the molecular formula as C_14_H_22_O_5_ and four indices of hydrogen deficiency. 14 carbon resonances can be observed from the ^13^C NMRspectrum of **3** (Table 2), corresponding to five methyls, two methylenes, two methines and five quaternary carbons. Accordingly, twenty-one of the hydrogens were carbon-bonded, so the remaining one could belong to a hydroxyl group. The ^13^C NMR spectrum displayed signals suggestive of a carbonyl carbon (*δ*_C_ 172.7) and four oxygenbearing carbons [*δ*_C_ 76.5 (CH), 83.6 (C), 84.7 (C) and 85.8 (C)]. The lack of any other olefinic carbon signals indicated that **3** contains three heterocycle rings, to fulfill its degrees of unsaturation. The ^1^H NMR(Table 2) spectrum of **3** recorded in C_5_D_5_N revealed the distinguished resonances of five methyl singlets (*δ*_H_ 1.22, 1.35, 1.40, 1.55, 1.83), two diastereotopic methylenes [(*δ*_H_ 2.30, 1H, m; 2.38, 1H, dd, *J* = 9.5, 2.1 Hz); and (*δ*_H_ 2.48, 1H, dd, *J* = 14.2, 3.9 Hz; 2.54, 1H, dd, *J* = 14.2, 5.8 Hz)], and two methines including one oxygenated (*δ*_H_ 4.37, dd, *J* = 5.7, 3.9 Hz) and one nonoxygenated (*δ*_H_ 2.32, m). The partial structure of **3** was established from a combination of HSQC, ^1^H–^1^H COSY, and HMBC experiments in C_5_D_5_N and CDCl_3_. HMBC correlations from H_3_-11 and H_3_-12 to C-7 and C-6, and from H_3_-10 to C-6, C-8, and C-9, corroborated the constitution of the lactone ring. HMBC cross-peaks of H-6 and H_2_-5 with C-4 confirmed the attendance of a five-membered heterocyclic ring B. HMBC data (correlations from H_3_-13 and H_3_-14 to C-1 and C-2, and from H-2 to C-3 and C-4) revealed the connectivity between C_1_–C_2_–C_3_–C_4_ as ring A (Fig. 8). Finally, correlations of H_2_-3 with C-5, and of H_2_-5 with C-3, implied that the C-4 tertiary carbon was a bridgehead between C-3 and C-5 as a spiroketal.

**Table 2:** ^1^H (500 MHz) and ^13^C (125 MHz) NMR data (C_5_D_5_N and CDCl_3_) of **3** (*δ* in ppm, *J* in Hz).

No	C_5_D_5_N		CDCl_3_	
	*δ*_C_	*δ*_H_*,* mult	*δ*_C_	*δ*_H_, mult
1	85.8		85.5	
2	76.5	4.37 dd (5.7, 3.9)	77.3	4.01 dd (5.8, 3.2)
3*α*	43.7	2.54 dd (14.2, 5.8)	43.0	2.25 dd (14.5, 5.9)
3*β*		2.48 dd (14.2, 3.9)		2.02^a^
4	110.6		110.0	
5*α*	32.4	2.30 m	32.2	2.01^a^
5*β*		2.38 dd (9.5, 2.1)		2.01^a^
6	47.0	2.32 m	47.3	2.03^a^
7	83.6		83.4	
8	84.7		84.5	
9	172.7		171.9	
10	22.7	1.83 s	22.9	1.65 s
11	23.8	1.35 s	24.3	1.41 s
12	29.5	1.22 s	30.0	1.34 s
13	27.5	1.40 s	27.0	1.21 s
14	23.0	1.55 s	22.3	1.21 s

^a^Overlapping signals.

**Figure 9:**
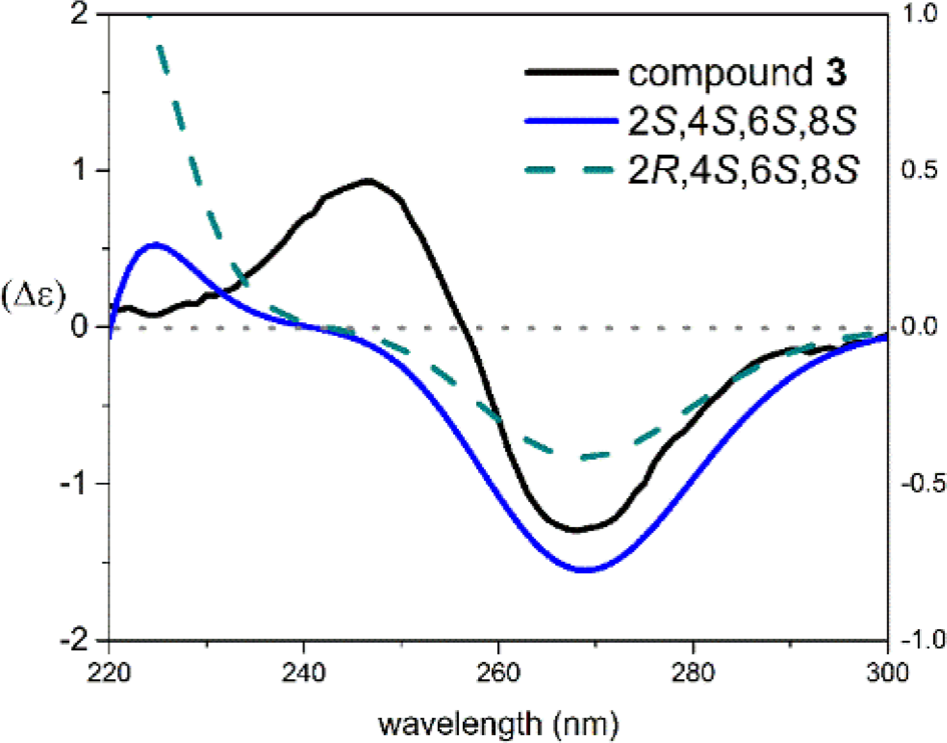
The experimental and TDDFT calculated ECD spectra of **3**.

The NOESY spectrum (Fig. 8) represented correlations between H-6, H_3_-10 and H_3_-11, as well as between H_3_-12 and H-5*β*, which emphasized the connection of rings B and C. Similarly, cross-peaks between H_3_-10 and H-3*α,* between H-5*α* and H-3*β*, between H-3*α* and H-2, as well as between H-3*β* and H_3_-13, were noticed and confirmed the linkage of rings A and B. The ECD spectrum of compound **3** showed a negative Cotton effect (CE) at 275 nm and a positive CE at 250 nm. Comparing the experimental ECD curve of **3** with calculated one resulted in deciphering the absolute stereochemistry of **3** as 2*S*, 4*S*, 6*S*, 8*S* (Fig. 9).

**Figure 10:**
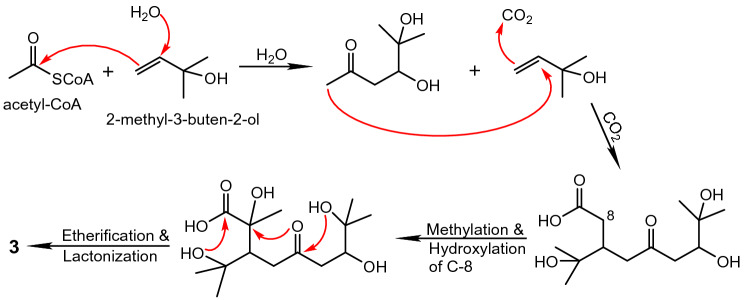
Putative biosynthetic pathway to **3**.

Isolation of **3** with intriguing 5,5-spiroketal lactone structure represents the first discovery from nature. From a biogenetic viewpoint, acetate and mevalonate pathways might be involved in the biosynthesis of this new scaffold. Although two isoprene C_5_ units appear to be involved in this process, it does not seem to fit the regular alkylation mechanism (Fig. 10).

Compounds **1**–**3** were examined for their in vitro antiprotozoal activity against the protozoan parasites *Trypanosoma brucei rhodesiense* and *Plasmodium falciparum*. Cytotoxicity was investigated in rat skeletal myoblast L6 cells and the selectivity indices (SI) for these compounds were calculated (Table 3).

**Table 3:** In vitro activity of compounds 1–3 against *T. b. rhodesiense* (STIB900) and *P*. *falciparum* (NF54) and cytotoxicity in L6 Cells.

Compound	IC_50_ (μM)^a^		
	*T. b. rhodesiense*	*P. falciparum*	L6 cells
1	3.07 ± 0.77; 11.07^b^	2.25 ± 0.10; 15.10^b^	33.98 ± 2.16
2	> 100.0	> 100.0	> 100.0
3	> 100.0	21.14 ± 1.60; 17.50^b^	> 100.0
Melarsoprol	0.04		
Chloroquine		0.01	
Podophyllotoxin			0.009

^a^Average of at least two independents assays.

^b^Selectivity index (SI): IC_50_ in L6 cells divided by IC_50_ in the titled parasitic strain.

Four known polycyclic polyprenylated acylphloroglucinols hyperibone A-C^15a^ and 7-*epi*-clusianone^25^, and two aromadendrane sesquiterpenoids lochmolin F^28^ and aromadendrane-4*β*,10*β*-diol^29^ were also isolated and identified in this study. Hyperibone A-C have been previously reported from the aerial parts of the plant, but the remainings were isolated for the first time from this species.

## Methods

### General experimental procedures

Optical rotation, UV and ECD spectra, and HR-ESIMS data were recorded as reported previously by us^30^. NMR experiments were measured on Bruker Avance III 500 and Bruker Avance II 600 spectrometers, using standard Bruker pulse sequences. HPLC separations were performed on a Knauer HPLC system according to our previous report^30^. Column chromatography (CC) was carried out on silica gel (70–230 mesh, Merck). TLC pre-coated silica gel F_254_ plates (Merck)) were used to detect and merge fractions; visualizing under UV light or by heating the plates after spraying with anisaldehyde-sulfuric acid reagent.

### Plant material

The aerial parts of *H. Scabrum* were gathered in the north of Iran in Yush village from Baladeh District, in June 2015. A voucher specimen was authenticated by Dr. Ali Sonboli and was deposited in the Herbarium of the Medicinal Plants and Drugs Research Institute, Shahid Beheshti University (MPH-2510).

### Extraction and isolation

After drying and powdering, the-aerial parts of *H. scabrum* (5.0 kg) were extracted (3 × 20 L, 24 h) with *n*-hexane, and the mixed extracts were condensed under vaccum to give a gummy residue (150 g). The dried extract was fractionated by open column chromatography on silica gel (1.0 kg, 5 × 100 cm, 70–230 mesh) eluted with a gradient of *n*-hexane–EtOAc (100:0 to 0:100), and then increasing amounts of MeOH (up to 50%). On the bases of TLC analysis, eight combined fractions (Fr.1-Fr.8) were obtained. From Fr.1 [eluted with *n*-hexane–EtOAc (95:5)] a yellow crude solid was obtained, which was triturated with MeOH to give 7-*epi*-clusianone (**4**) as white powder (100 mg). Fr.2 (1.0 g) was further purified on silica gel column chromatography (200 g, 2 × 60 cm) and eluted with *n*-hexane-CH_2_Cl_2_-(CH_3_)_2_CO (58:40:2) to get four subfractions (Fr.2.1-Fr.2.4). Fr.2.2 (70 mg) was subjected to silica gel column (70 g, 1 × 50 cm) eluted with *n*-hexane-(CH_3_)_2_CO (90:10) to afford compound **1** (7.0 mg). Fr.2.3 (150 mg) was submitted to a silica gel column (140 g, 1.5 × 70 cm), eluted with *n*-hexane-(CH_3_)_2_CO (80:20) to obtain two subfractions (Fr.2.3a-Fr.2.3b). Repeated purification of Fr.2.3a (80 mg) by silica gel CC [100 g, 1 × 70 cm, eluted with CH_2_Cl_2_-(CH_3_)_2_CO (97:3)], afforded hyperibone A (2.5 mg), hyperibone B (1.5 mg) and hyperibone C (14 mg). Fr.4 (1.9 g) was further separated via silica gel column chromatography (300 g, 4 × 60 cm) using CH_2_Cl_2_-(CH_3_)_2_CO (95:5) as eluent to produce five subfractions (Fr.4.1-Fr.4.5). Fr.4.1 (170 mg) was further chromatographed on silica gel (150 g, 1.5 × 80 cm) using CH_2_Cl_2_-1-propanol-MeOH (95:4:1) as mobile phase to yield lochmolin F (5.7 mg) and aromadendrane-4*β*,10*β*-diol (2 mg). Fr.4.3 (100 mg) was also separated on silica gel (120 g, 1.5 × 50 cm) and eluted with *n*-hexane-(CH_3_)_2_CO (75:25) to obtain compound **3** (3.9 mg). Fr 7 (2.1 g) was subsequently chromatographed on a silica gel CC (300 g, 4 × 60 cm) eluted with *n*-hexane-(CH_3_)_2_CO (65:35) and followed by increasing concentrations of 1-propanol (2%) to give four subfractions (Fr.7.1–Fr.7.4). Fr.7.3 (400 mg) was applied to a silica gel column (100 g, 2.0 × 80 cm) and eluted with *n*-hexane-CHCl_3_-MeOH (47:47:6) to give six subfractions (Fr.7.3.1–Fr.7.3.6). Fr.7.3.6 (40 mg) was separated by preparative reversed-phase HPLC using the mobile phase of MeCN-H_2_O (55:45, v/v) to yield compound **2** (1.6 mg).

- **Compound 1** Yellow gum, [α] ^25^_D_ + 17.2 (*c* = 1.0, CH_3_OH); HR-ESIMS [M + H]^+^ *m/z* 535.3067 (calcd for C_33_H_43_O_6_, 535.3054); ^1^H and ^13^C NMR data in Table 1.
- **Compound 2** White powder, [α] ^25^_D_ + 30.5 (*c* = 1.0, CHCl_3_); HR-ESIMS [M + H]^+^ *m/z* 413.1941 (calcd for C_24_H_29_O_6_, 413.1959); ^1^H and ^13^C NMR data in Table 1.
- **Compound 3** Colorless oil, [α] ^25^_D_ + 9.1 (*c* = 1.0, CH_3_OH); HR-ESIMS [M + Na]^+^ *m/z* 293.1366 (calcd for C_14_H_22_O_5_Na, 293.1365); ^1^H and ^13^C NMR data in Table 2.

### X-ray crystallographic analysis of 2

C_24_H_28_O_6_, colorless prism, 0.20 × 0.08 × 0.05 mm, space group P2_1_2_1_2_1_, orthogonal, *a* = 6.8847(4) Å, *b* = 15.1910(16) Å, *c* = 20.6691(14) Å, *V* = 2161.7(3) Å^3^, *Z* = 4, ρ_calcd_ = 1.267 g·cm^‒3^, T = 173 K, Cu radiation (λ = 1.54184 Å), θ range 3.6°‒62.1°, 5988 reflections collected, 3323 independent, R_int_ = 0.0708, 308 parameters, wR2 = 0.1110 (all data), R1 = 0.0512 [*I* > 2σ(*I*)], absolute structure parameter 0.0(2). The crystal data of compound **2** was placed in the Cambridge Crystallographic Data Centre (CCDC 1992711).

### ECD calculations of 1 and 3

Conformational analysis of compounds **1** and **3** was carried out on MacroModel 9.1 software by applying OPLS-2005 force field in H_2_O (Schrödinger LLC) with the Gaussian 09 program package^31^, and ECD curves were obtained using SpecDis version1.64^32^.

### In vitro antiprotozoal assay

The in vitro inhibitory activities of compounds **1**–**3** were evaluated against the protozoan parasites *T. b. rhodesiense* (STIB900) trypomastigotes and *P*. *falciparum* (NF54) LEF stage and cytotoxicity in L6 cells (rat skeletal myoblasts) by adopting the same procedures as the reported previously^33,34^.

## Supplementary Information

Supplementary Information.
