# Supplementary material for: Unusual derivatives from Hypericum scabrum
Source: Sci Rep. 2021 Jan 14;10:22181. doi: 10.1038/s41598-020-79305-y (PMC7809121; doi:10.1038/s41598-020-79305-y)
Supplement: Supplementary file 1 — Supplementary Information. [file 41598_2020_79305_MOESM1_ESM.pdf]

Unusual Derivatives from *Hypericum scabrum*

Sara Soroury[1,2], Mostafa Alilou[3], Thomas Gelbrich[4], Marzieh Tabefam[2], Ombeline Danton[5], Samad N. Ebrahimi[2], Marcel Kaiser[6], Matthias Hamburger[5], Hermann Stuppner[3] & Mahdi Moridi Farimani[2*]

[1] Department of Phytochemistry, Faculty of Science, Golestan University, Gorgan 15759-49138, Iran. [2] Department of Phytochemistry, Medicinal Plants and Drugs Research Institute, Shahid Beheshti University, G. C., Evin, Tehran, Iran. [3] Institute of Pharmacy/Pharmacognosy, Center for Molecular Biosciences Innsbruck, University of Innsbruck, Innrain 80/82, Innsbruck 6020, Austria. [4] Institute of Pharmacy, Pharmaceutical Technology, University of Innsbruck, Innrain 52c, 6020 Innsbruck, Austria. [5] Division of Pharmaceutical Biology, University of Basel, Klingelbergstrasse 50, 4056 Basel, Switzerland. [6]Swiss Tropical and Public Health Institute, Socinstrasse 57, CH-4002 Basel, Switzerland.

*email: m_moridi@sbu.ac.ir

Supporting information

Contents

1D and 2D NMR spectra of compound 1 in $CDCl_3$......4
Figure S1. ^1H NMR spectrum (500 MHz) of compound 1 in $CDCl_3$. 4
Figure S2. Expanded ^1H NMR spectrum (500 MHz) of compound 1 in $CDCl_3$......4
Figure S3. DEPT-Q spectrum (125 MHz) of compound 1 in $CDCl_3$. 5
Figure S4. Expanded DEPT-Q spectrum (125 MHz) of compound 1 in $CDCl_3$......5
Figure S5. HSQC-DEPT spectrum of compound 1 in $CDCl_3$......6
Figure S6. ^1H-^1H COSY spectrum of compound 1 in $CDCl_3$. 6
Figure S7. HMBC spectrum of compound 1 in $CDCl_3$......7
Figure S8. Expanded HMBC spectrum of compound 1 in $CDCl_3$. 7
Figure S9. NOESY spectrum of compound 1 in $CDCl_3$. 8
Figure S10. Expanded NOESY spectrum (500 MHz) of compound 1 in CDCl3. 8
Figure S11. HRMS spectrum of compound 1. 9
1D and 2D NMR spectra of compound 2 in $CDCl_3$......10
Figure S12. ^1H NMR spectrum (600 MHz) of compound 2 in $CDCl_3$. 10
Figure S13. Expanded ^1H NMR spectrum (600 MHz) of compound 2 in $CDCl_3$......10
Figure S14. ^{13}C NMR spectrum (150 MHz) of compound 2 in $CDCl_3$. 11
Figure S15.Expanded 13C NMR spectrum (150 MHz) of compound 2 in CDCl3......11
Figure S16. HSQC spectrum of compound 2 in $CDCl_3$......12
Figure S17. ^1H-^1H COSY spectrum of compound 2 in $CDCl_3$. 12
Figure S18. HMBC spectrum of compound 2 in $CDCl_3$......13
Figure S19. NOESY spectrum of compound 2 in $CDCl_3$. 13
Figure S20. HRMS spectrum of compound 2. 144
1D and 2D NMR spectra of compound 3 in $CDCl_3$ and C_5D_5N......15
Figure S21. ^1H NMR spectrum (500 MHz) of compound 3 in $CDCl_3$. 155
Figure S22. DEPT-Q spectrum (125 MHz) of compound 3 in $CDCl_3$. 155
Figure S23. HSQC-DEPT spectrum of compound 3 in $CDCl_3$......166
Figure S24. ^1H-^1H COSY spectrum of compound 3 in $CDCl_3$. 166
Figure S25. HMBC spectrum of compound 3 in $CDCl_3$......17

Figure S26. NOESY spectrum of compound 3 in $CDCl_3$. 17
Figure S27. ^{1}H NMR spectrum (500 MHz) of compound 3 in C_5D_5N. 18
Figure S28. DEPT-Q spectrum (125 MHz) of compound 3 in C_5D_5N. 18
Figure S29. HSQC-DEPT spectrum of compound 3 in C_5D_5N. 19
Figure S30. ^{1}H-^{1}H COSY spectrum of compound 3 in C_5D_5N. 19
Figure S31. HMBC spectrum of compound 3 in C_5D_5N. 20
Figure S32. NOESY spectrum of compound 3 in C_5D_5N. 20
Figure S33. HRMS spectrum of compound 3. 21
Table S4. Energies and Cartesian Coordinates (Å) for the Optimized Structure of compound **1**…22
Table S5. Energies and Cartesian Coordinates (Å) for the Optimized Structures of compound **3**…23

1D and 2D NMR spectra of compound 1 in CDCl3

Figure S1. ^{1}H NMR spectrum (500 MHz) of compound 1 in CDCl3.

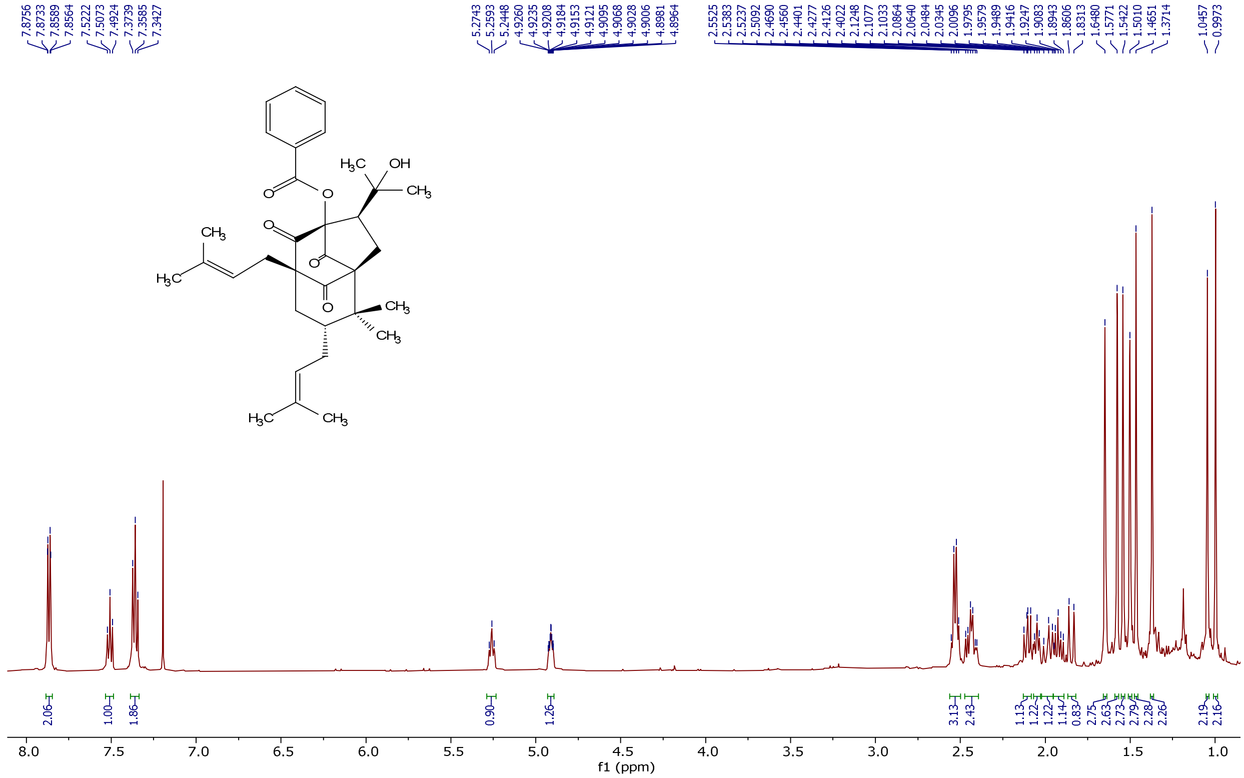

Figure S2. Expanded ^{1}H NMR spectrum (500 MHz) of compound 1 in CDCl3.

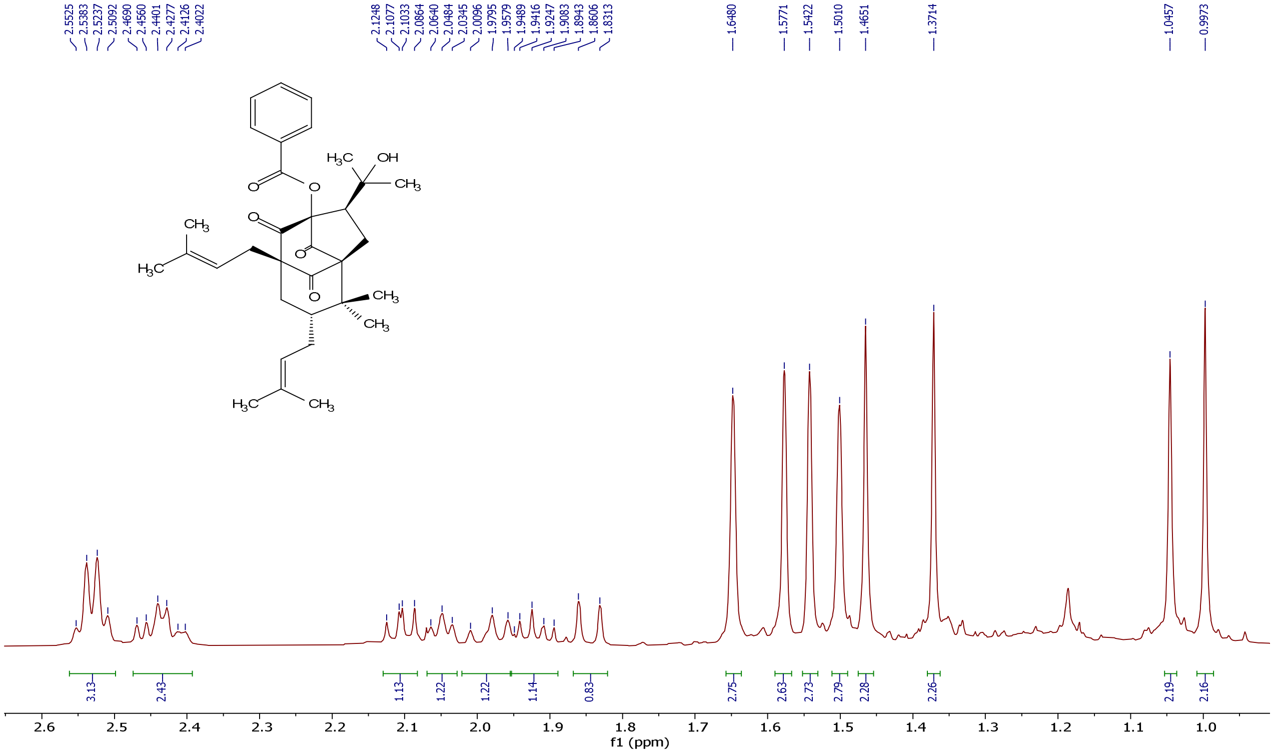

Figure S3. DEPT-Q spectrum (125 MHz) of compound 1 in $CDCl_3$.

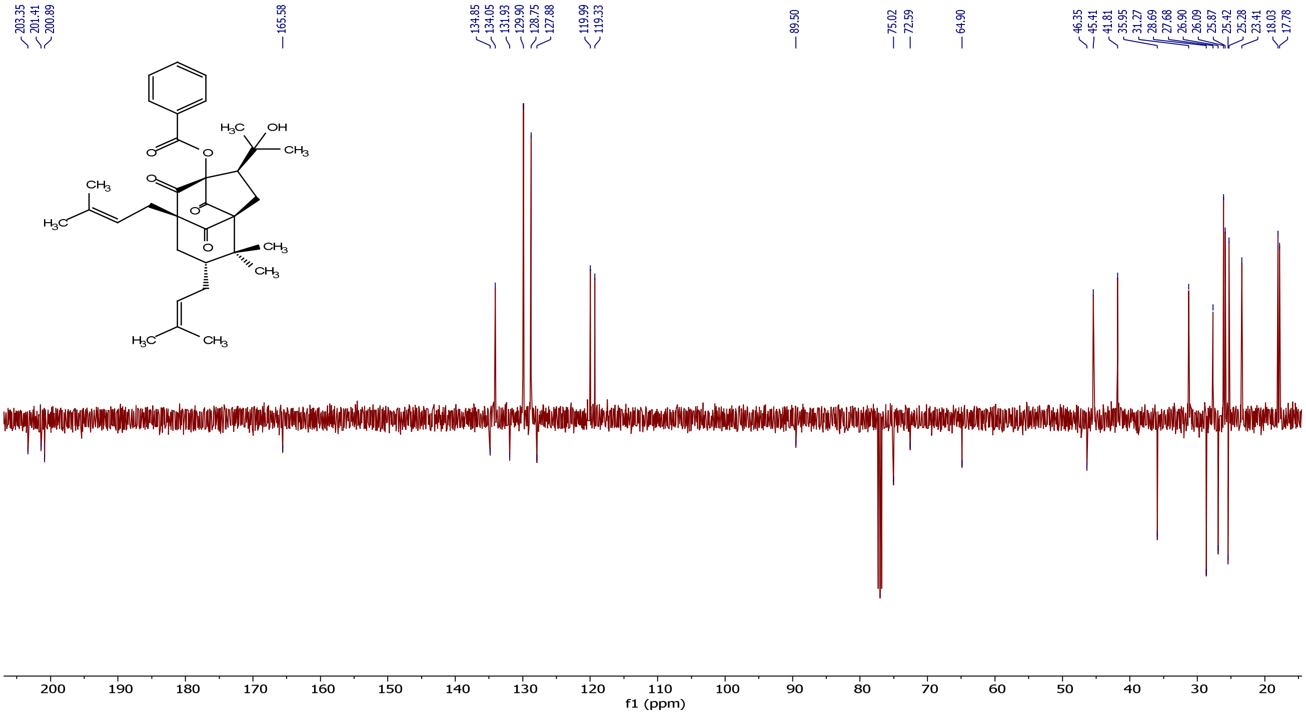

Figure S4. Expanded DEPT-Q spectrum (125 MHz) of compound 1 in $CDCl_3$.

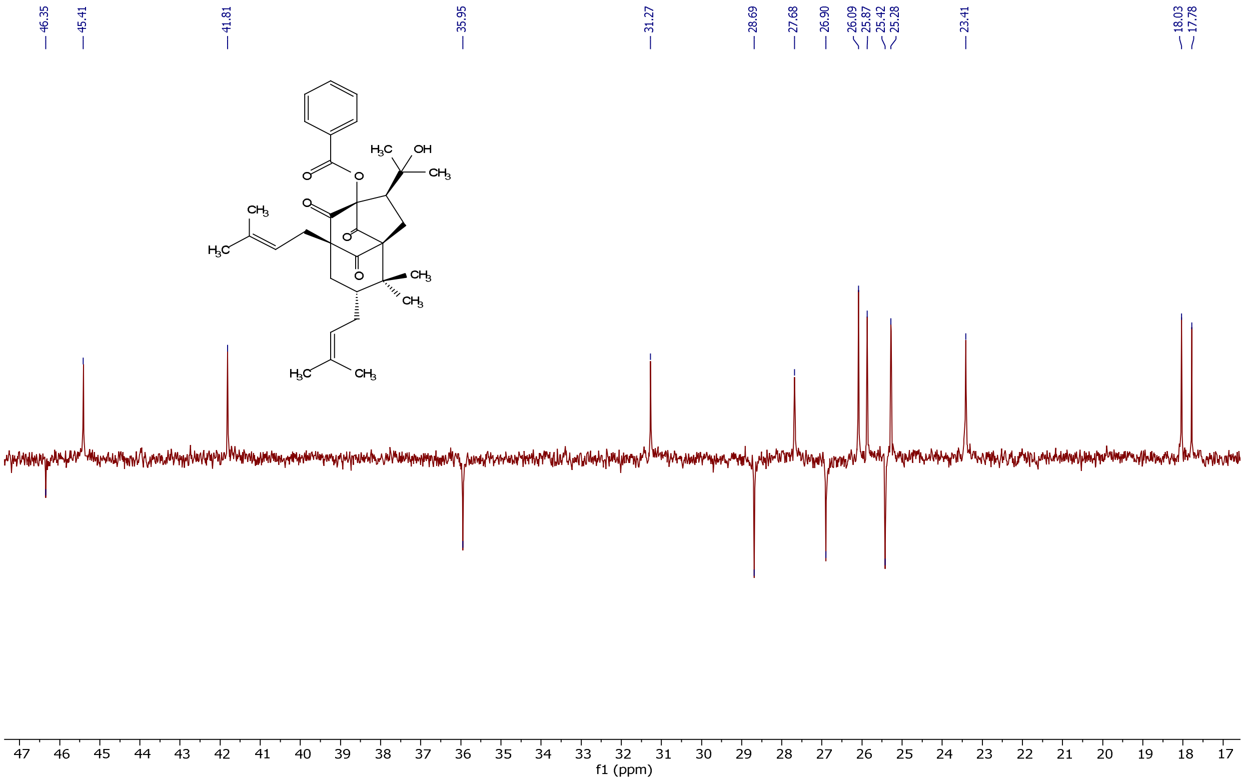

Figure S5. HSQC-DEPT spectrum of compound 1 in $CDCl_3$.

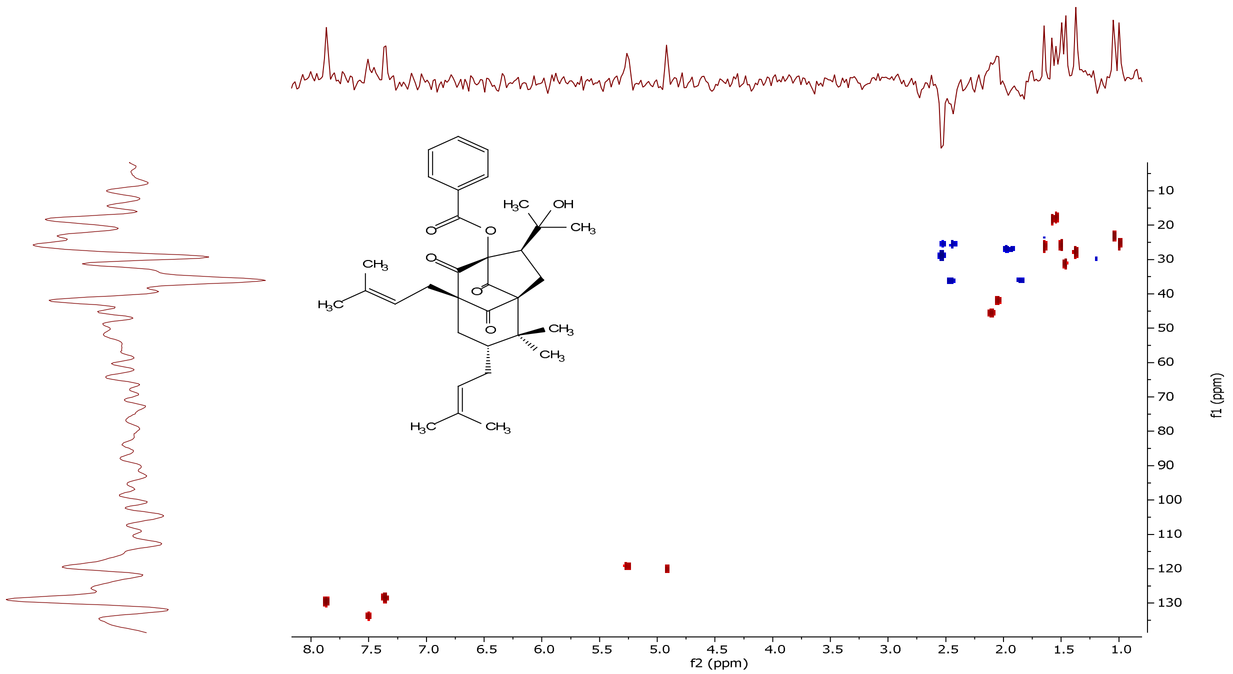

Figure S6. ^{1}H-^{1}H COSY spectrum of compound 1 in $CDCl_3$.

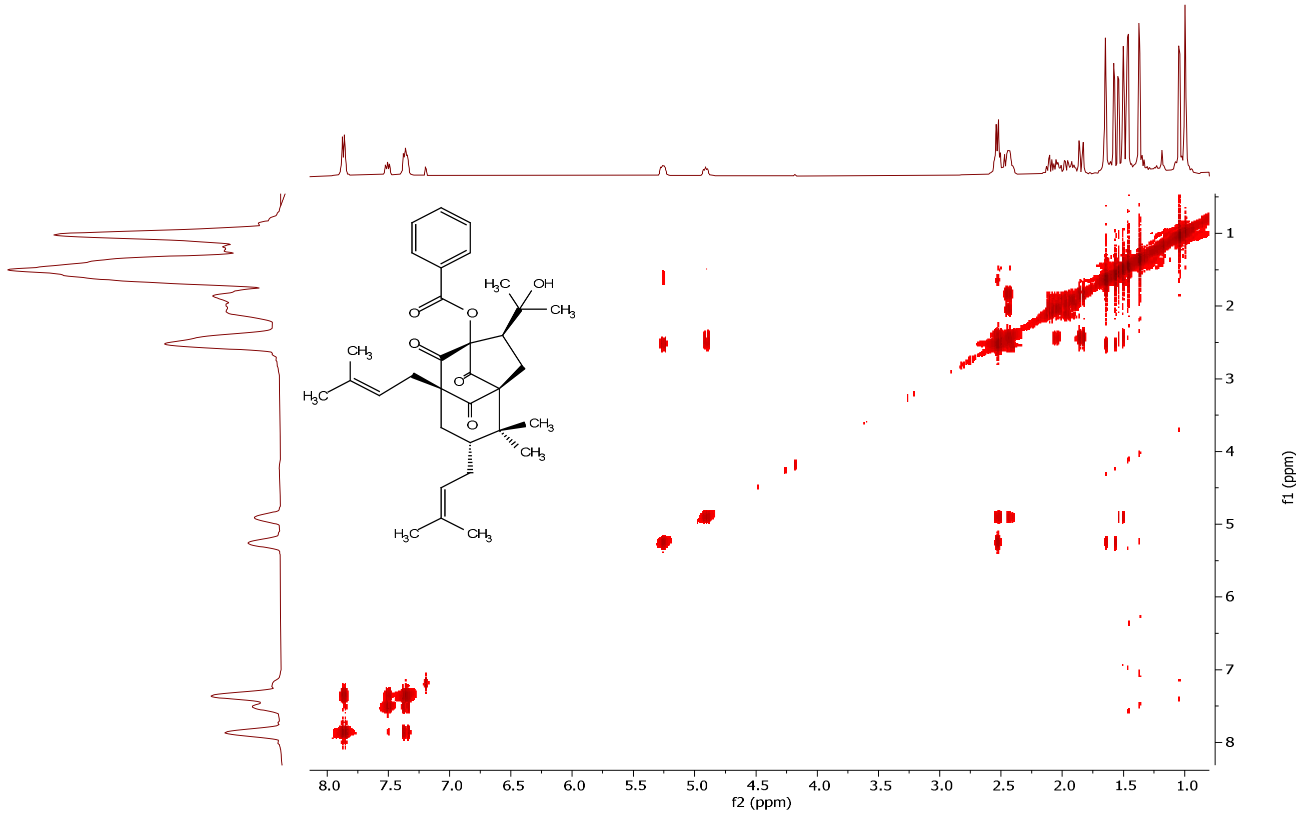

Figure S7. HMBC spectrum of compound 1 in CDCl3.

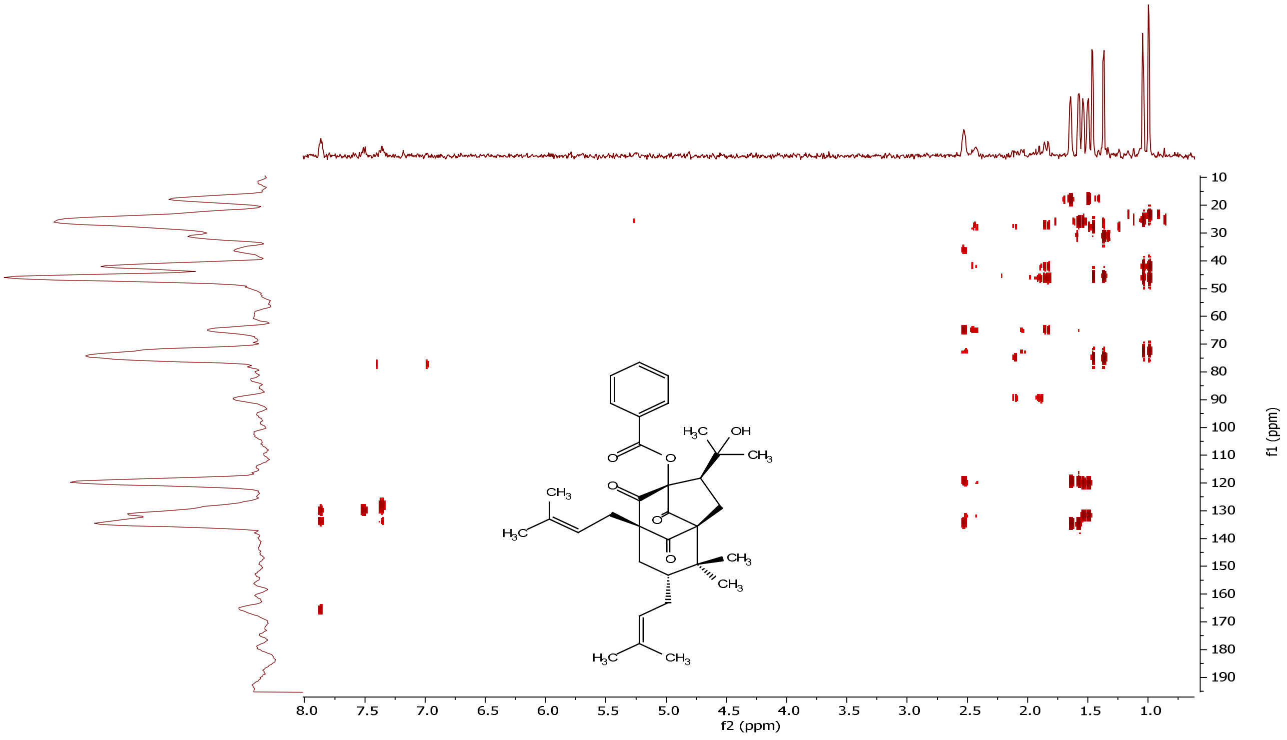

Figure S8. Expanded HMBC spectrum of compound 1 in CDCl3.

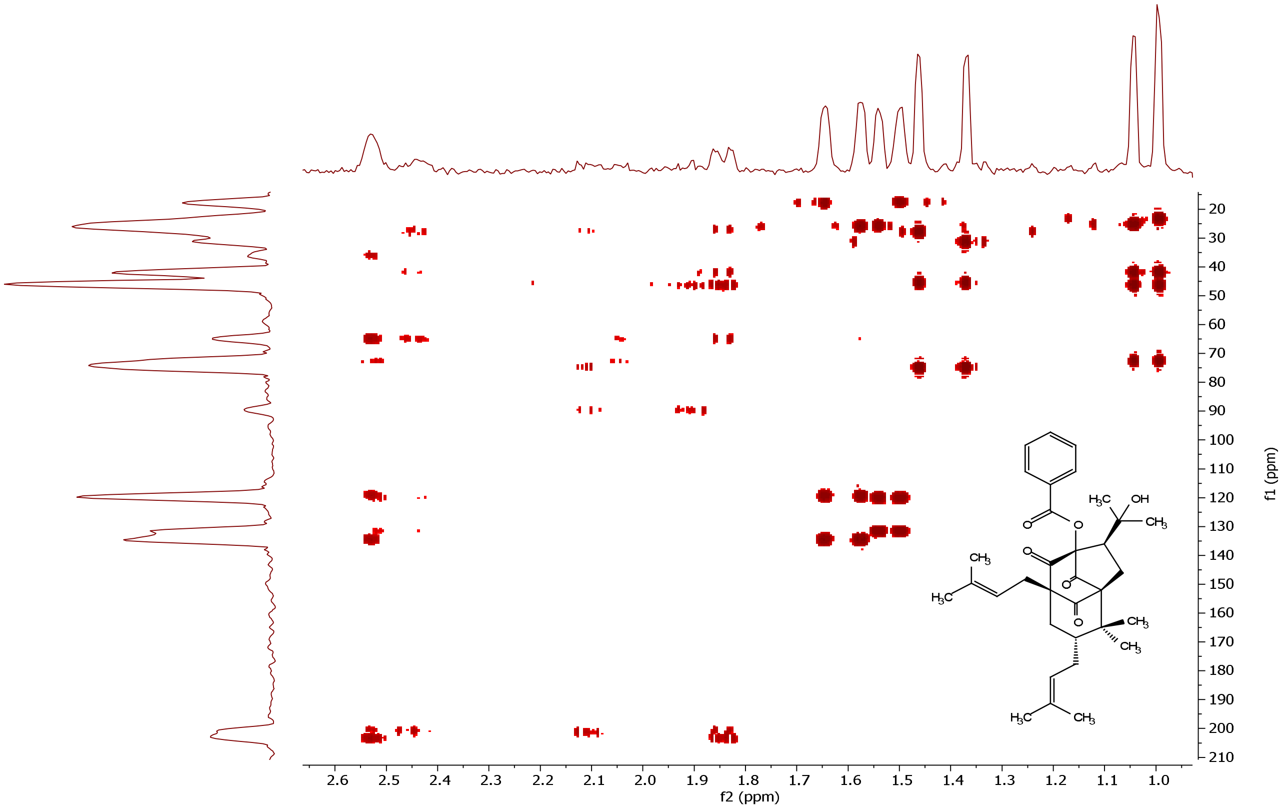

Figure S9. NOESY spectrum of compound 1 in CDCl₃.

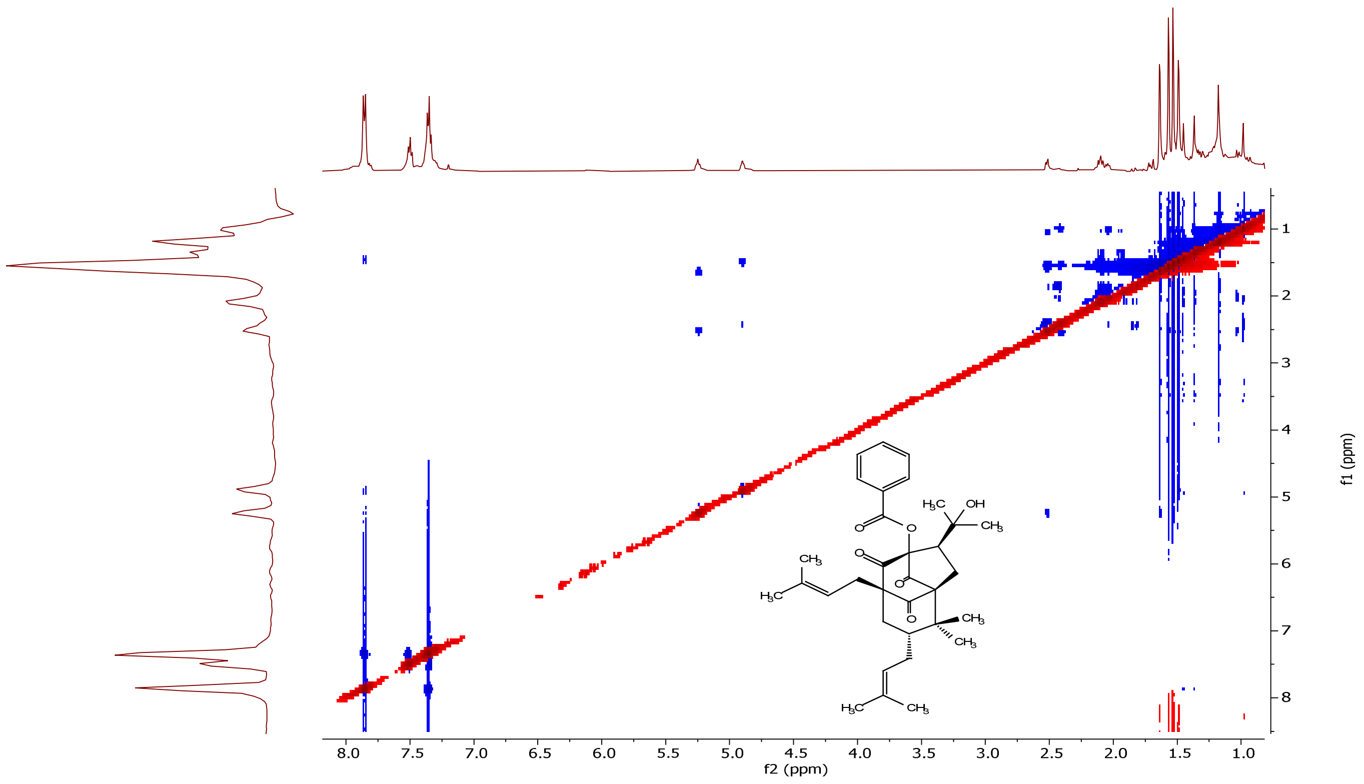

Figure S10. Expanded NOESY spectrum (500 MHz) of compound 1 in CDCl3.

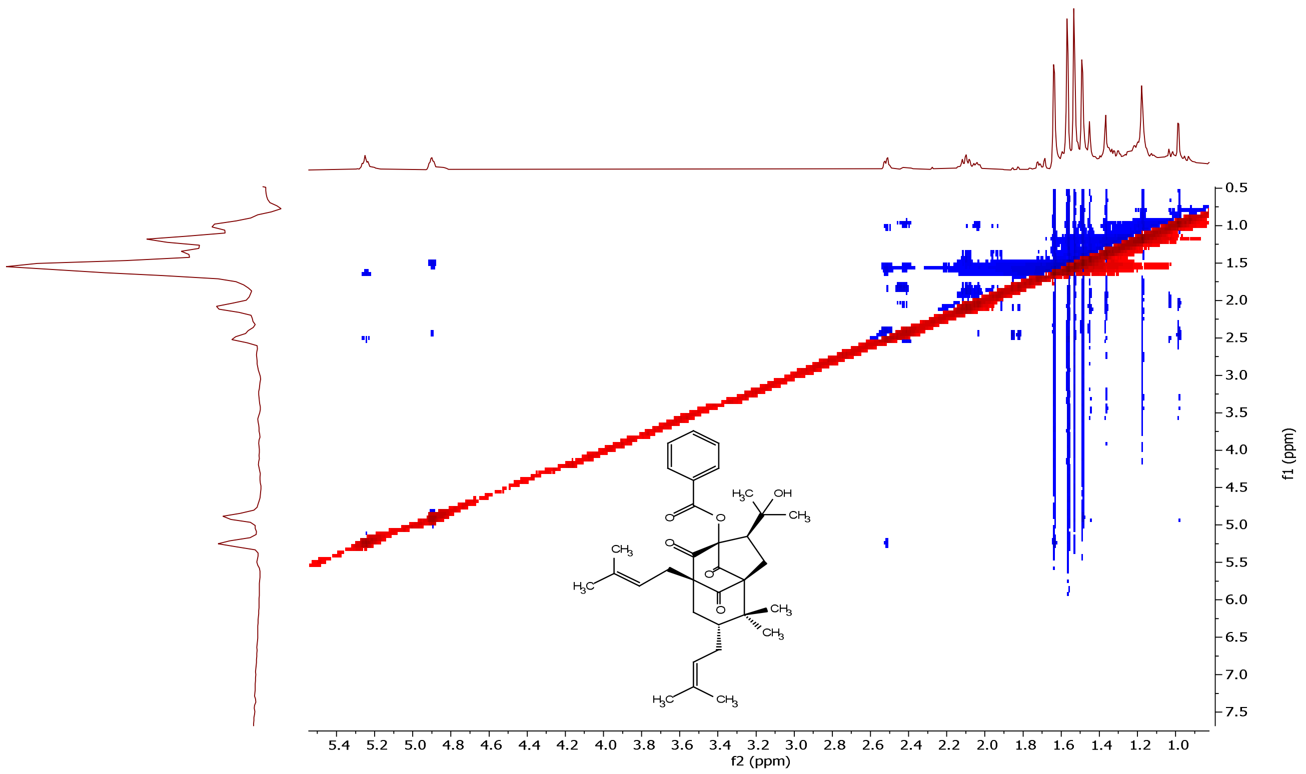

Figure S11. HRMS spectrum of compound 1.

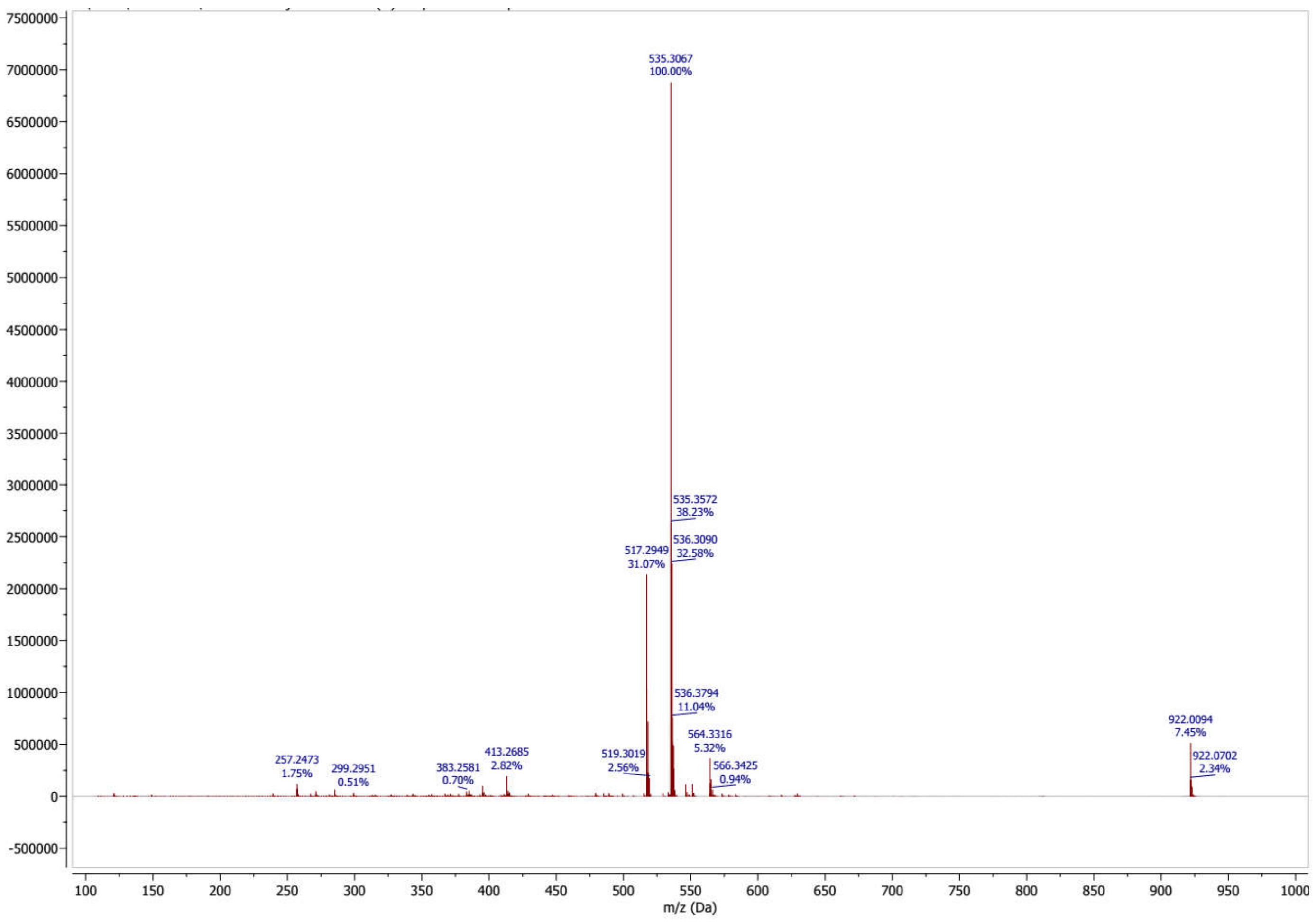

1D and 2D NMR spectra of compound 2 in $CDCl_3$

Figure S12. ^{1}H NMR spectrum (600 MHz) of compound 2 in $CDCl_3$

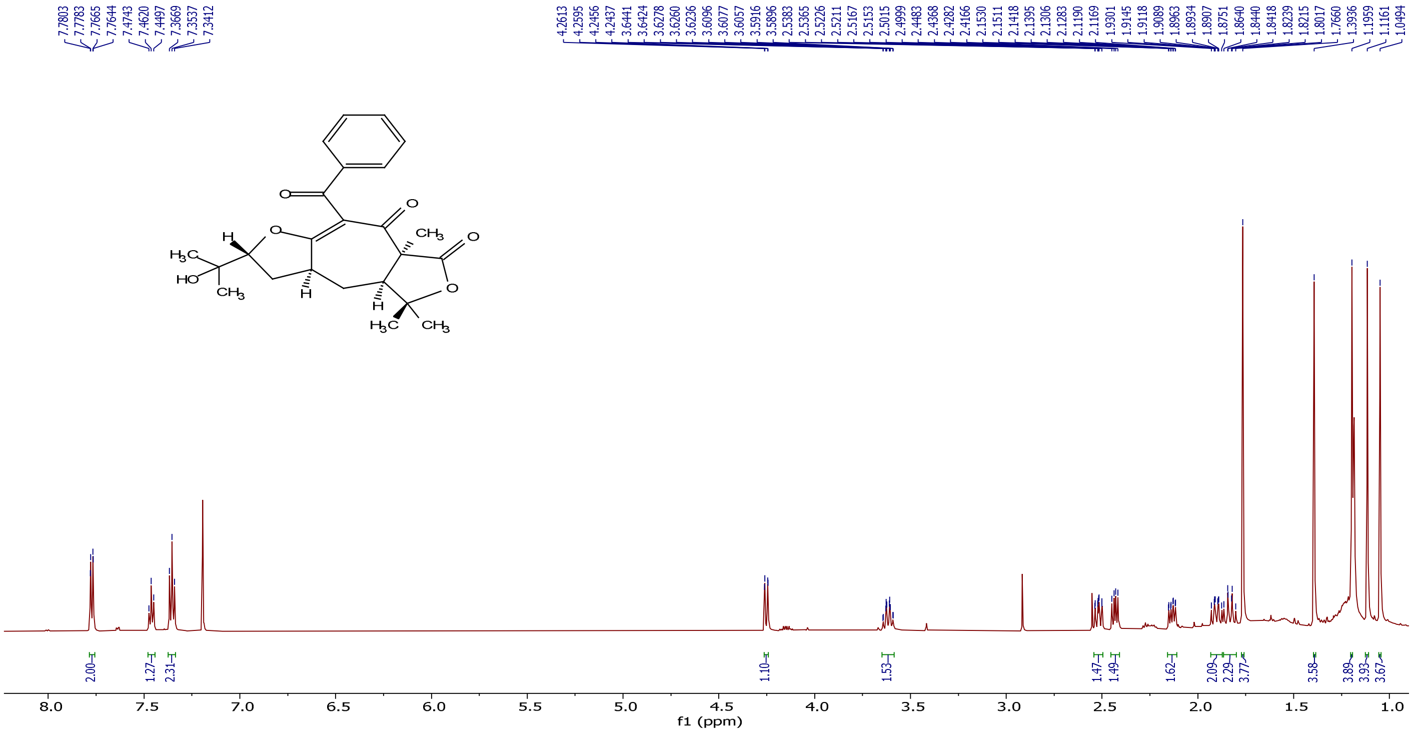

Figure S13. Expanded ^{1}H NMR spectrum (600 MHz) of compound 2 in $CDCl_3$

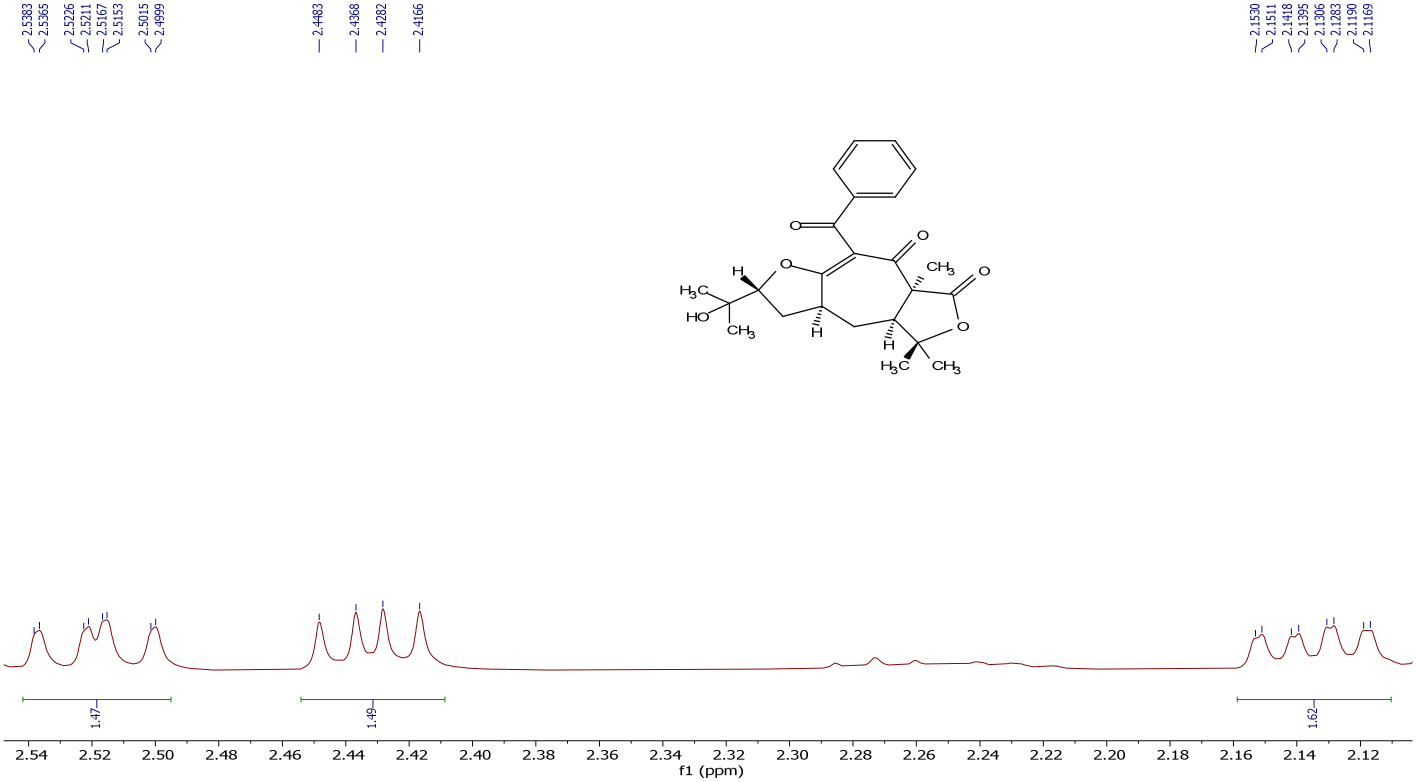

Figure S14. ^{13}C NMR spectrum (150 MHz) of compound 2 in $CDCl_3$.

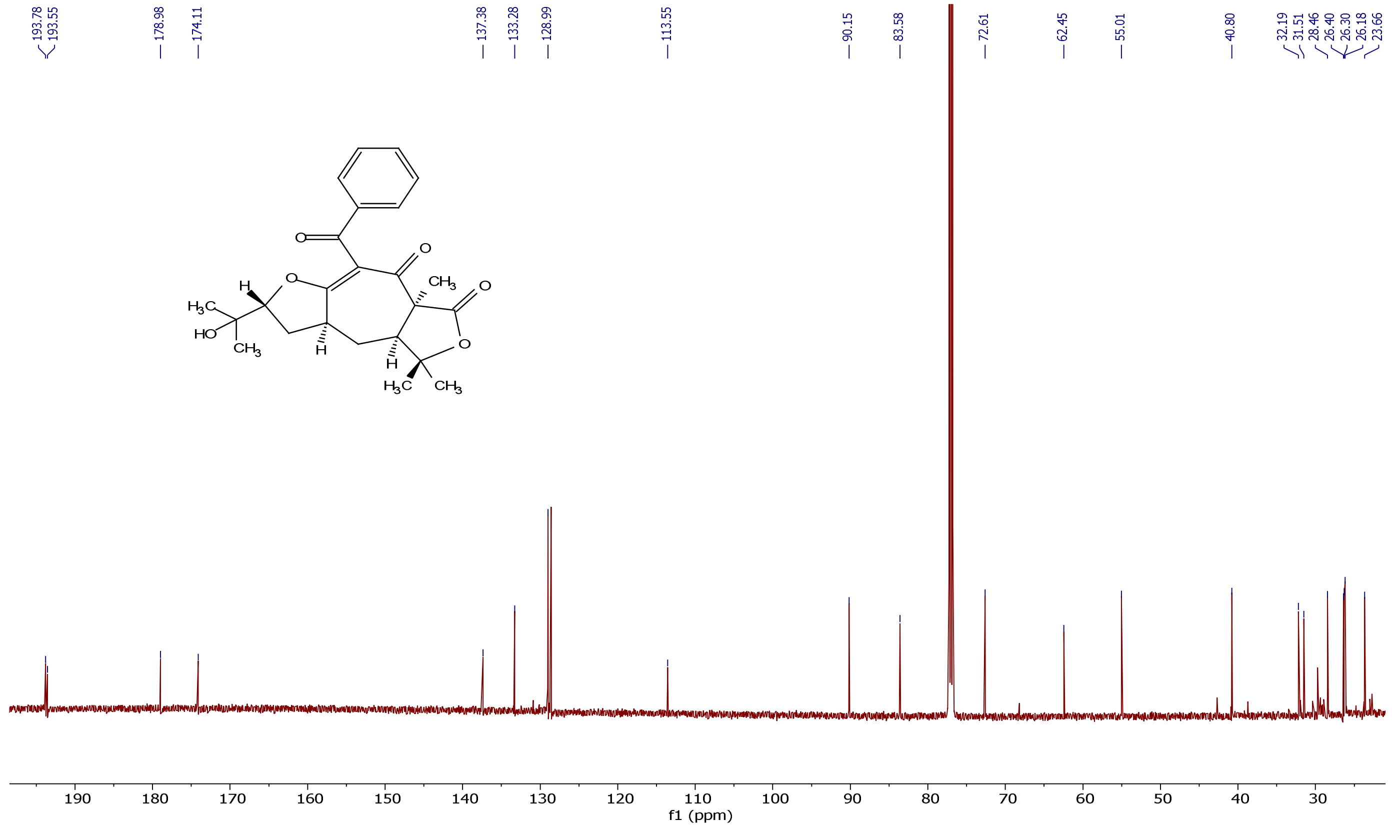

Figure S15.Expanded ^{13}C NMR spectrum (150 MHz) of compound 2 in $CDCl_3$.

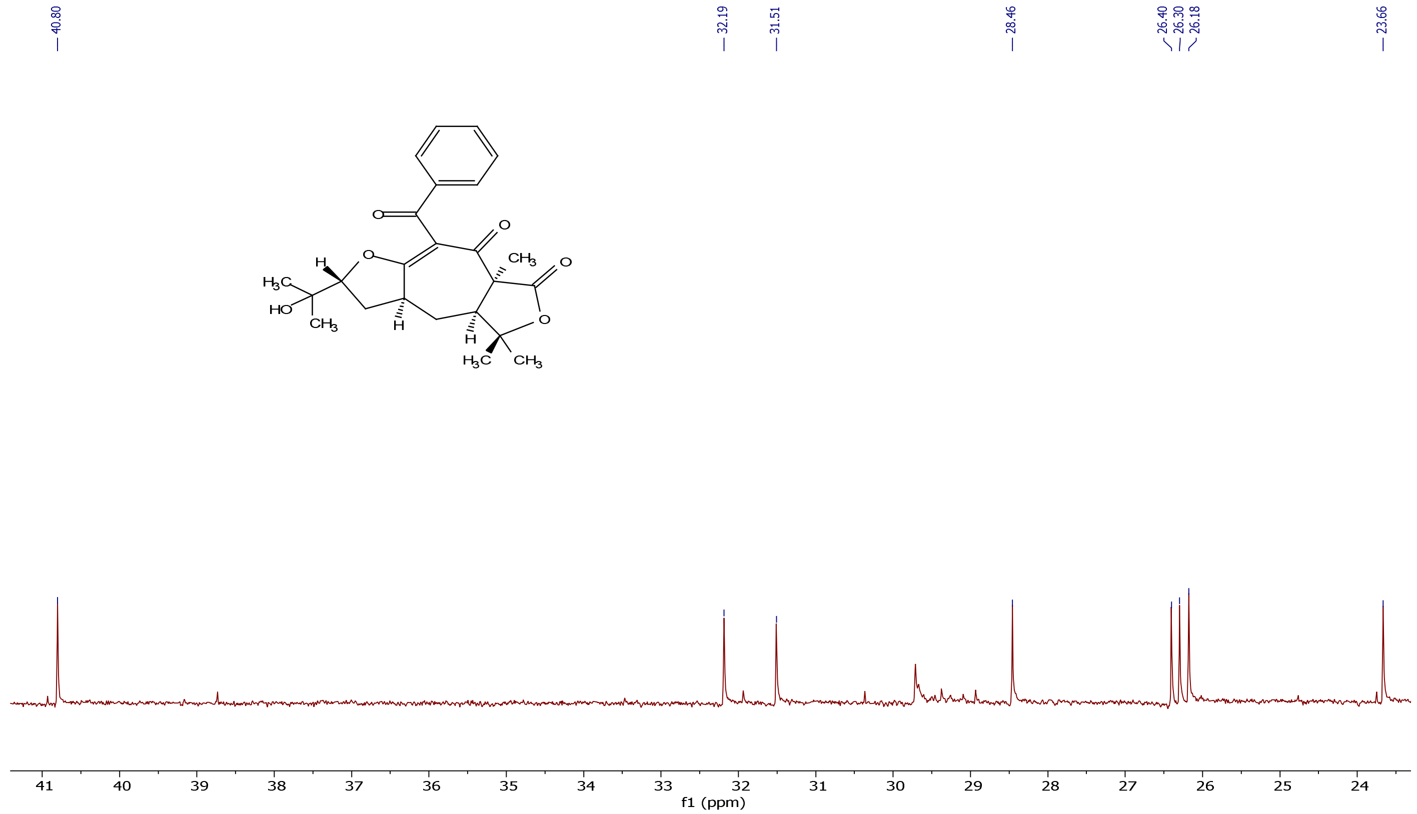

Figure S16. HSQC spectrum of compound 2 in CDCl₃.

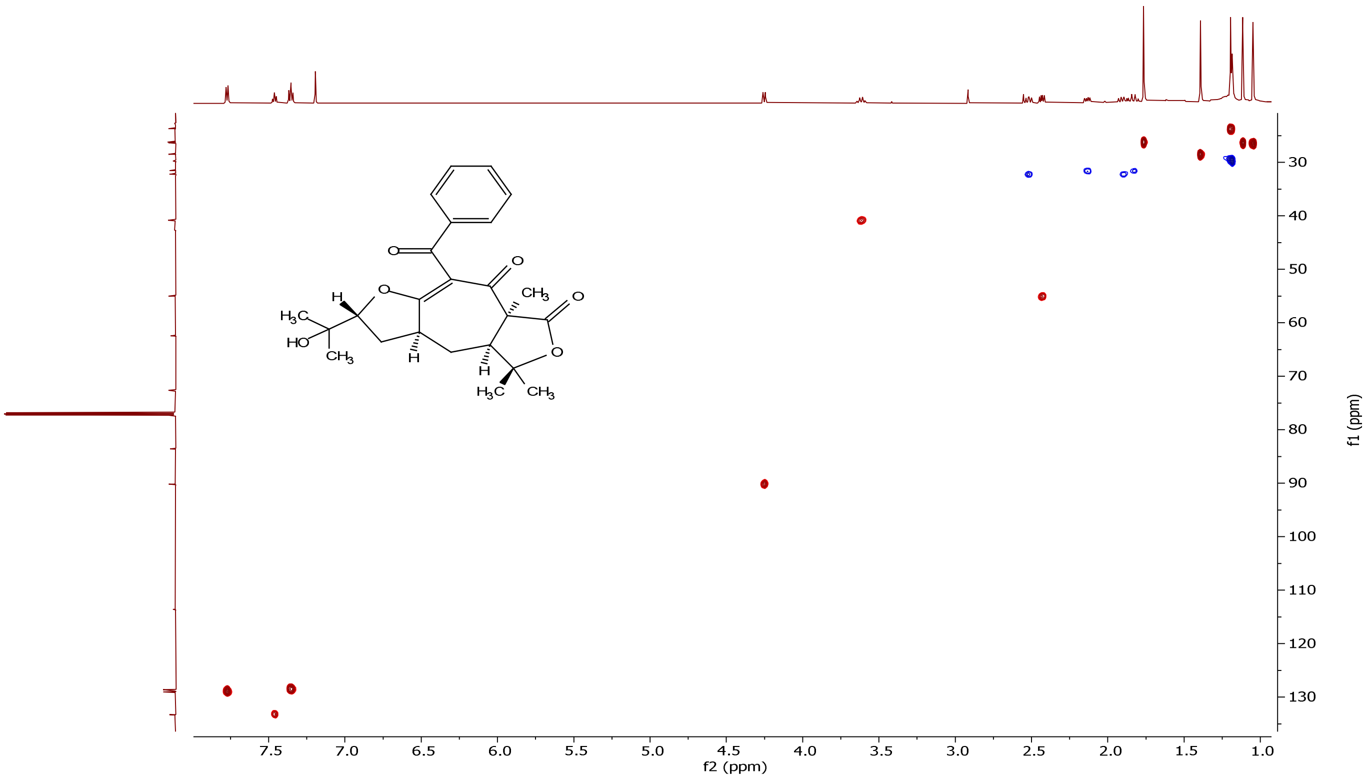

Figure S17. ^{1}H-^{1}H COSY spectrum of compound 2 in CDCl₃.

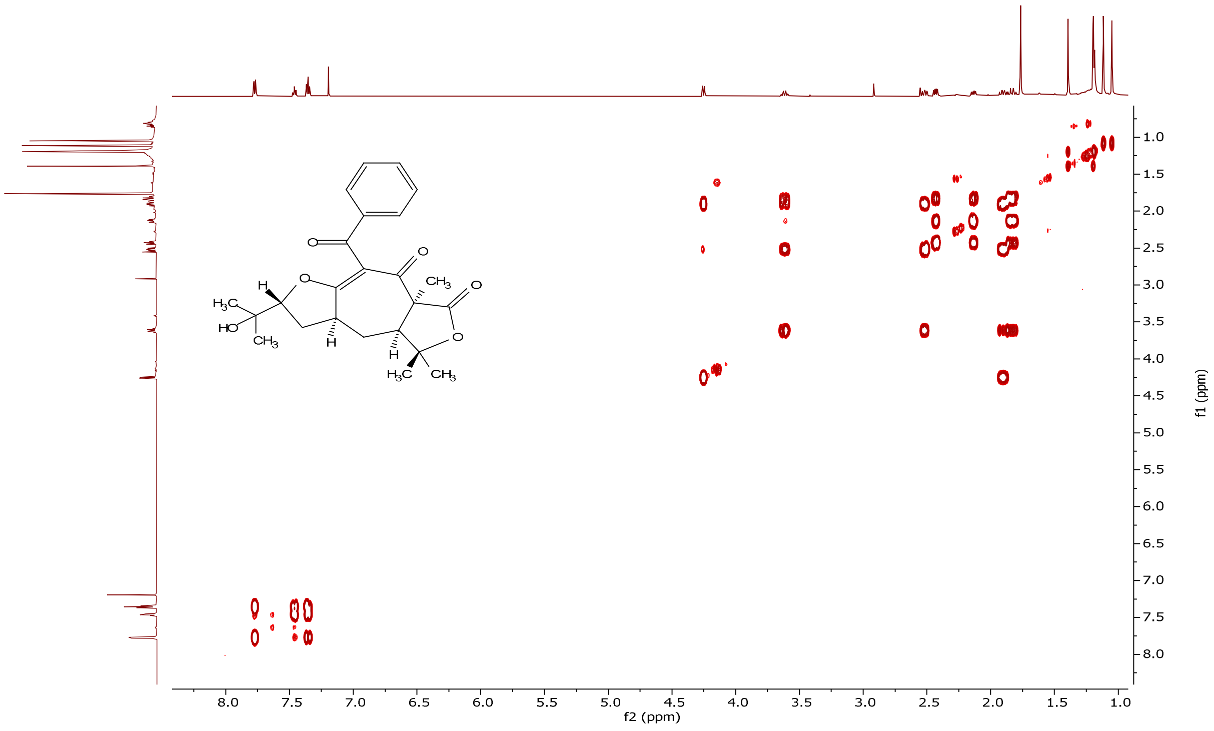

Figure S18. HMBC spectrum of compound 2 in CDCl₃.

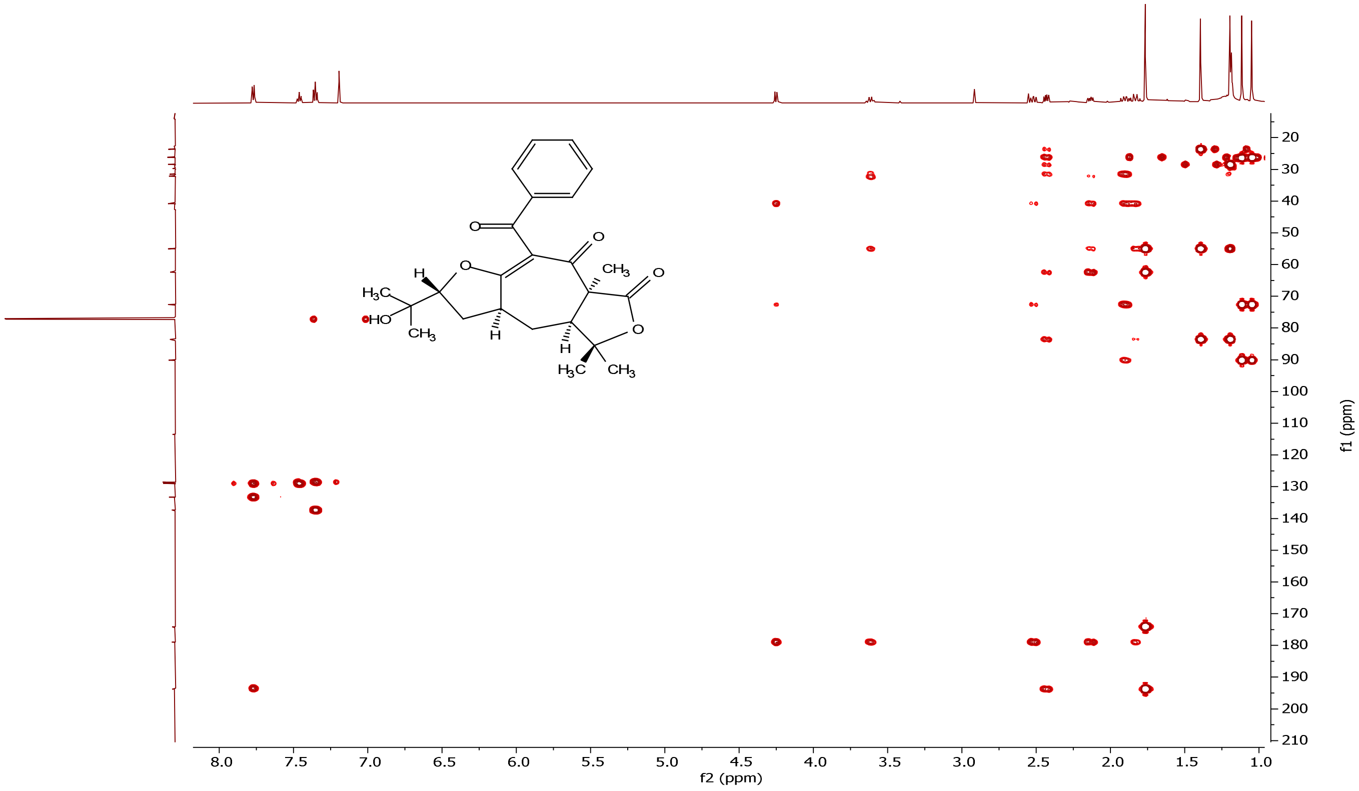

Figure S19. NOESY spectrum of compound 2 in CDCl₃.

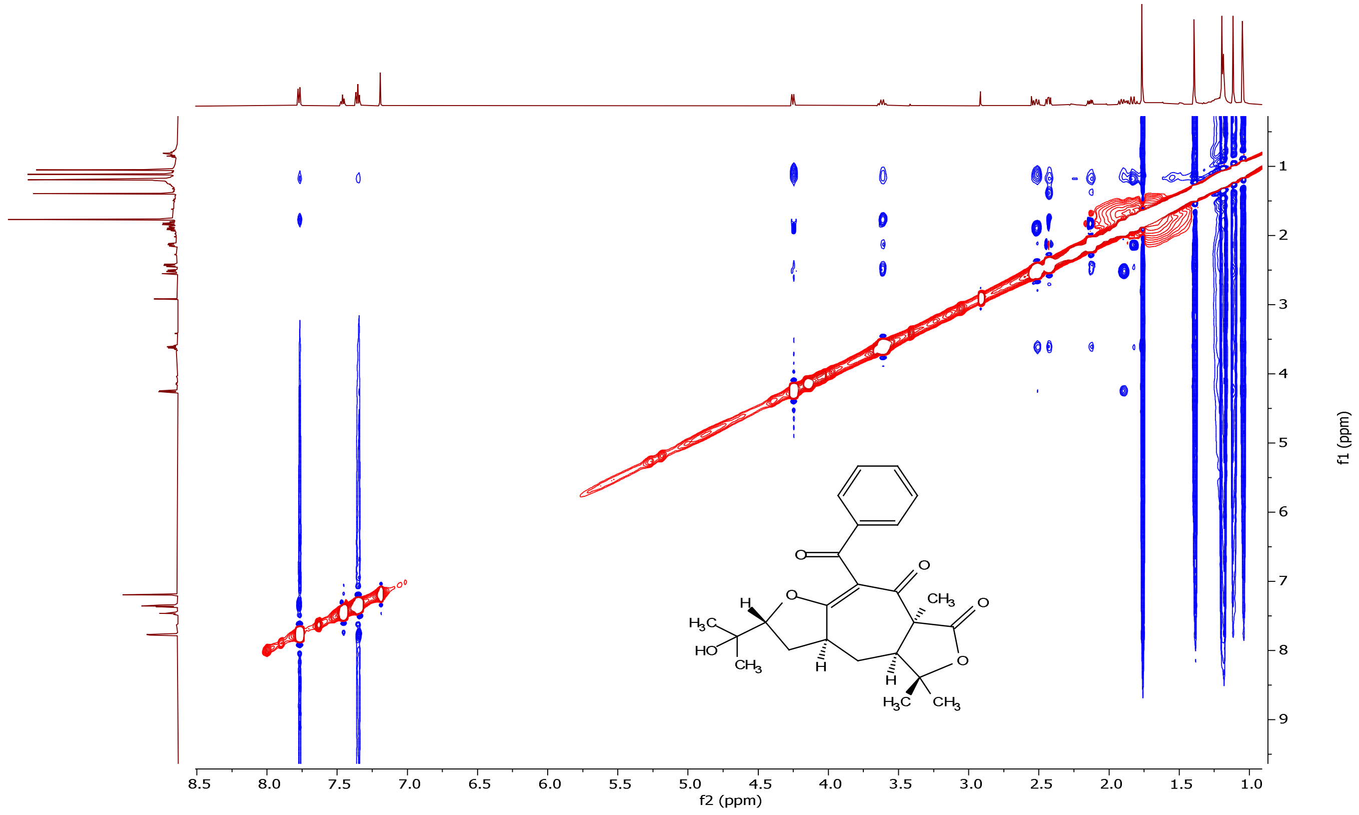

Figure S20. HRMS spectrum of compound 2.

Mass Spectrum SmartFormula Report

Analysis Info

Analysis Name	D:\Data\Pharmakognosie\Mostafa\pure_compounds_LCMS\HI2_run_1_2_01_26031.d	Acquisition Date	12/10/2019 12:59:27 PM
Method	screen_pos_2019.m	Operator	Simon
Sample Name	HI2_run_1	Instrument / Ser#	micrOTOF-Q II 10202
Comment	#7 - oeliger A. 1200784/0 Aceton		

Acquisition Parameter

Source Type	ESI	Ion Polarity	Positive	Set Nebulizer	23.2 psi
Focus	Not active	Set Capillary	4500 V	Set Dry Heater	220 °C
Scan Begin	200 m/z	Set End Plate Offset	-500 V	Set Dry Gas	6.0 l/min
Scan End	1500 m/z	Set Collision Cell RF	452.4 Vpp	Set Divert Valve	Source

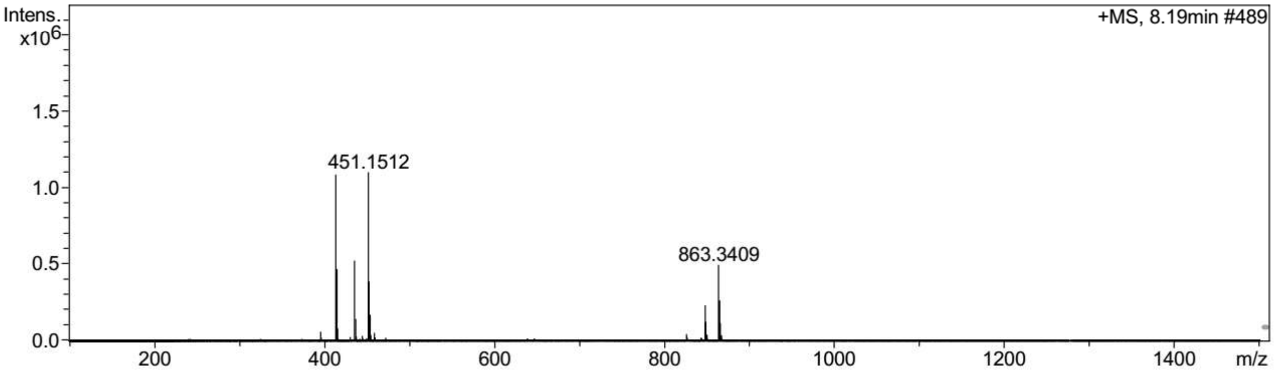

Meas. m/z	#	Formula	Score	m/z	err [mDa]	err [ppm]	mSigma	rdb	e⁻ Conf	N-Rule
413.1941	1	C 24 H 29 O 6	100.00	413.1959	1.8	4.3	99.3	10.5	even	ok

1D and 2D NMR spectra of compound 3 in $CDCl_3$ and C_5D_5N

Figure S21. ^1H **NMR spectrum (500 MHz) of compound 3 in** $CDCl_3$**.**

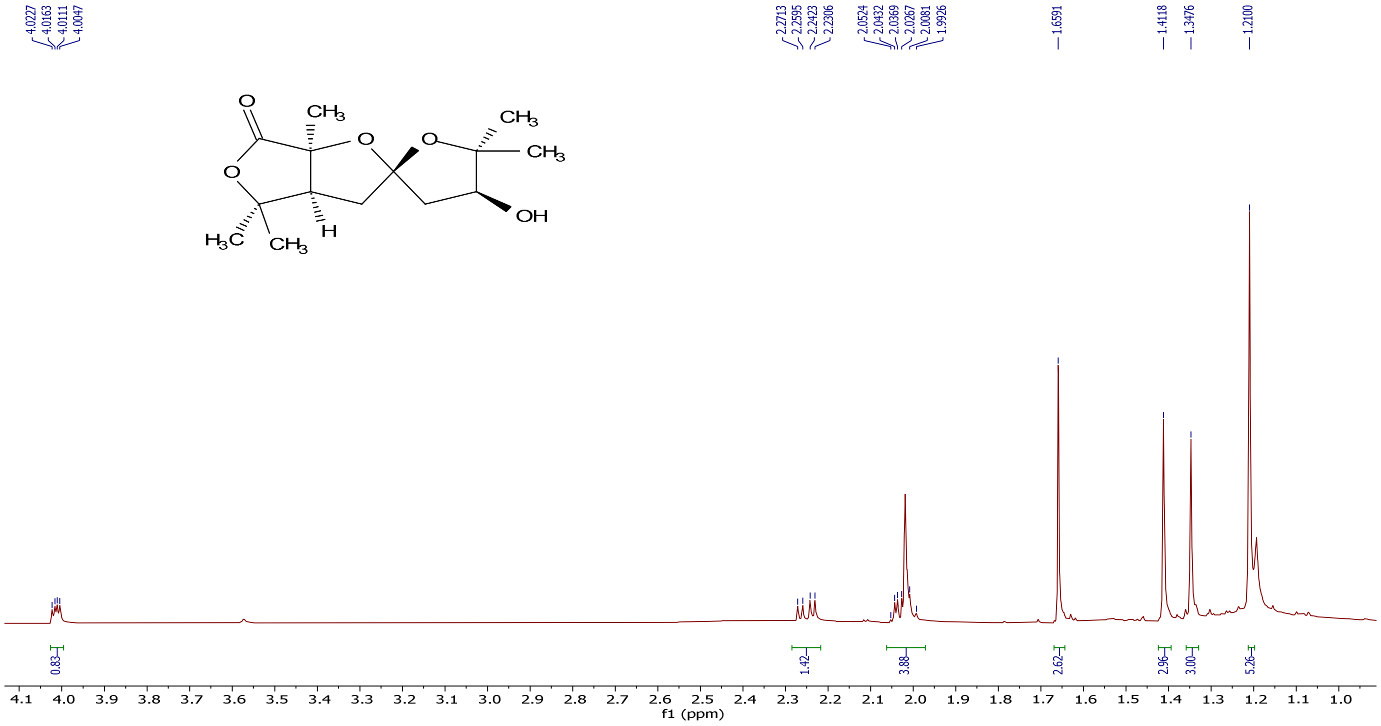

Figure S22. DEPT-Q spectrum (125 MHz) of compound 3 in $CDCl_3$**.**

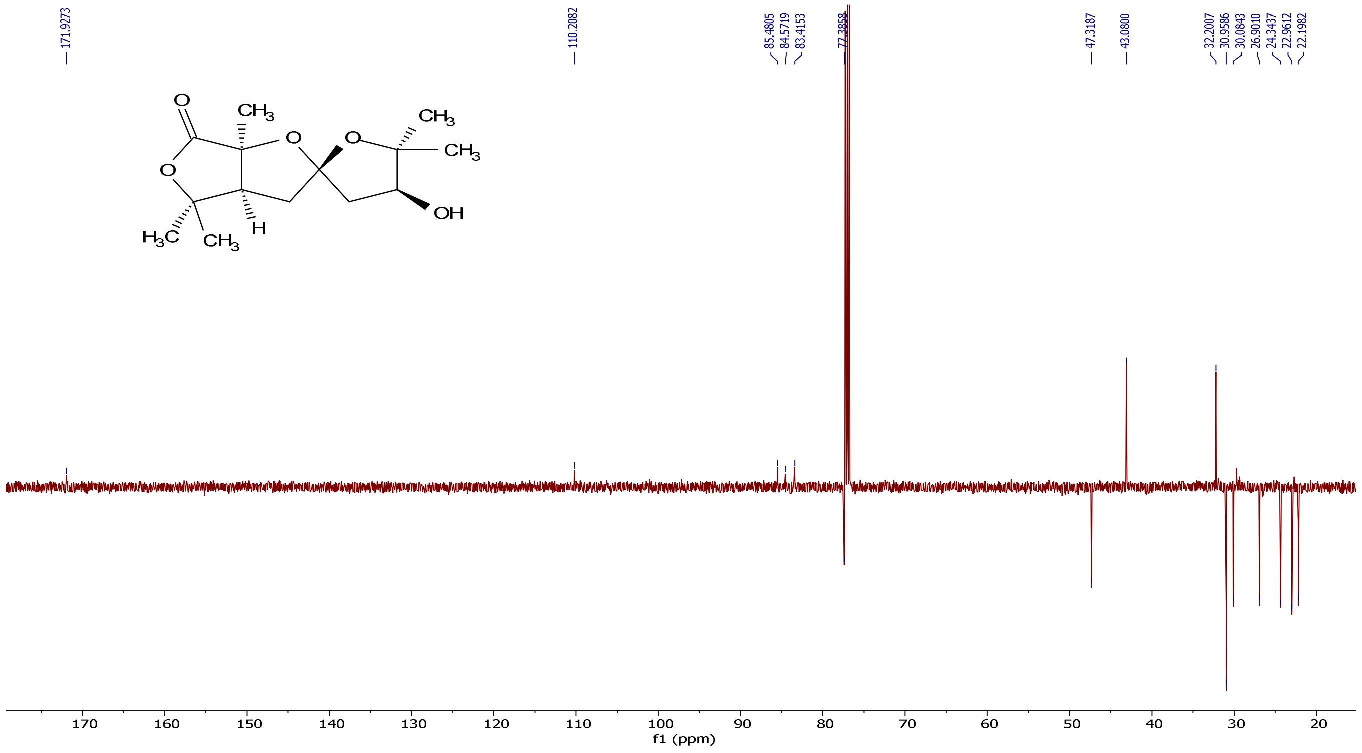

Figure S23. HSQC-DEPT spectrum of compound 3 in CDCl₃.

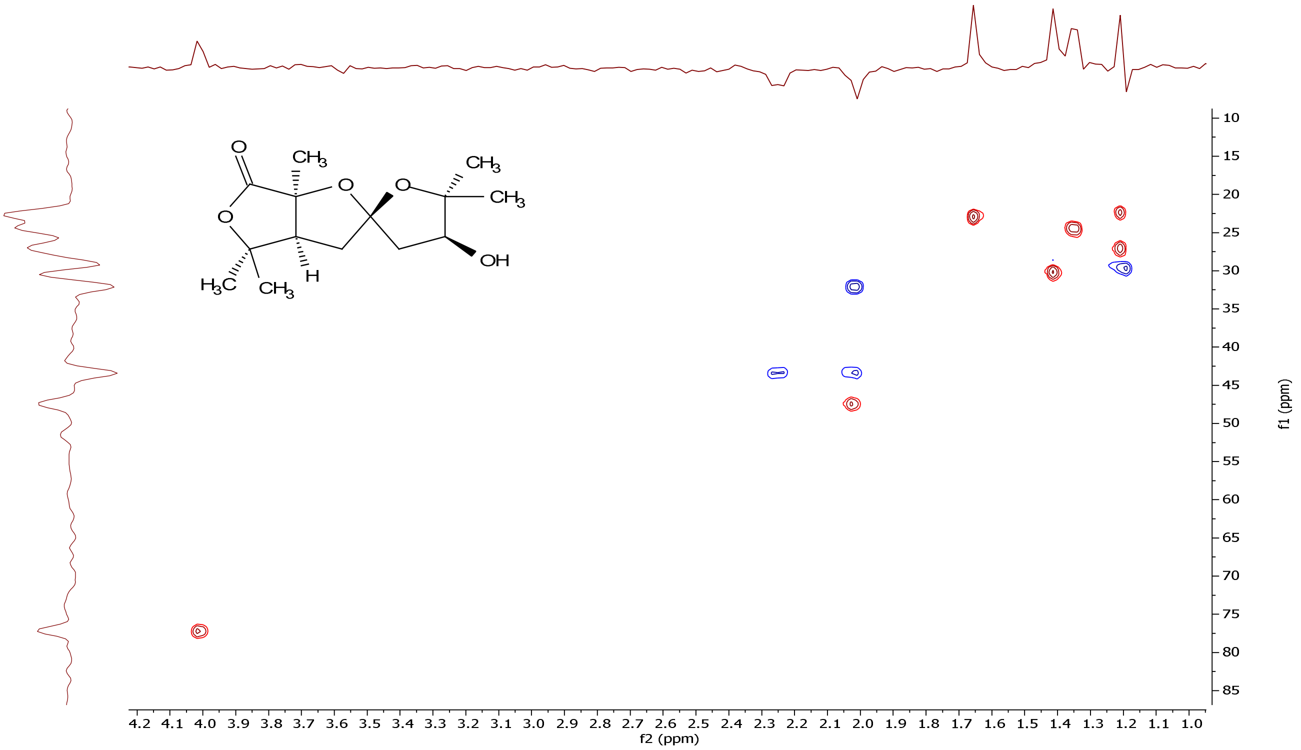

Figure S24. ^{1}H-^{1}H COSY spectrum of compound 3 in CDCl₃.

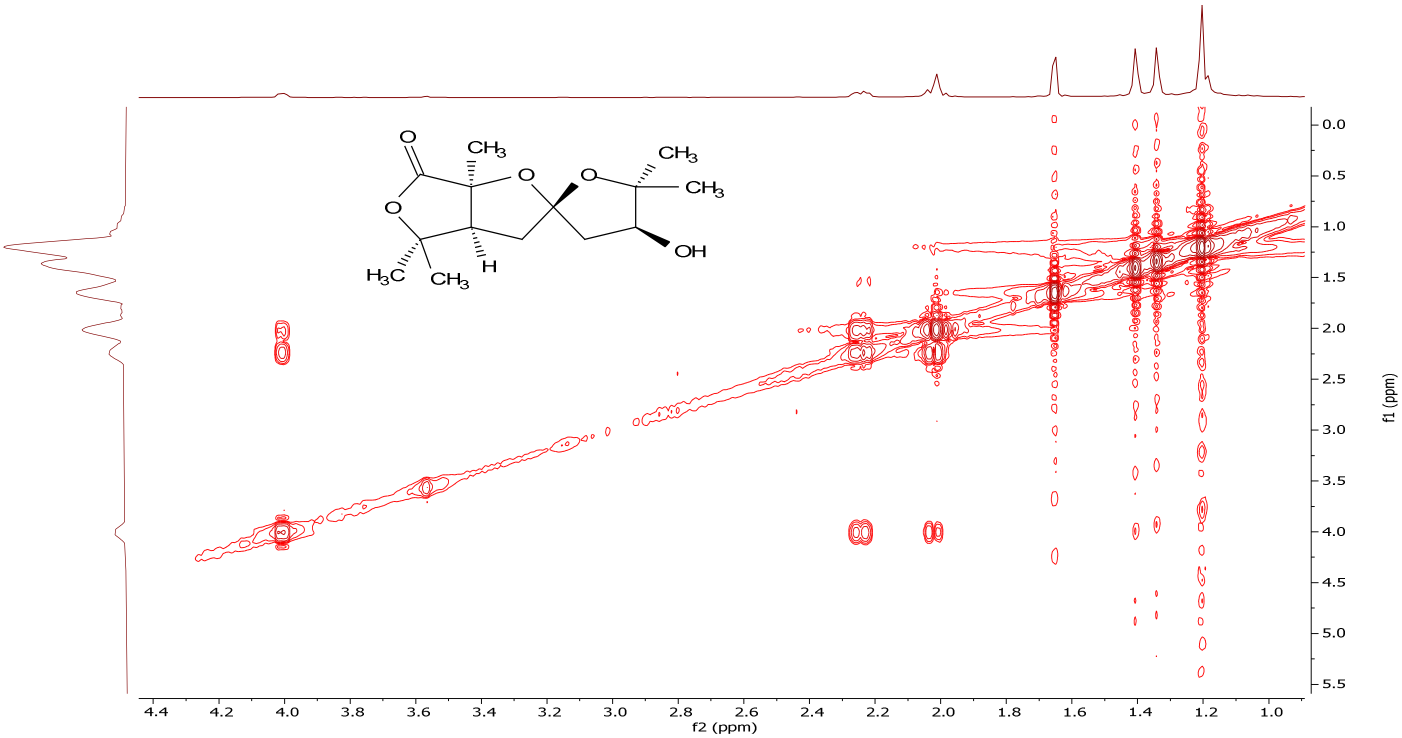

Figure S25. HMBC spectrum of compound 3 in CDCl₃.

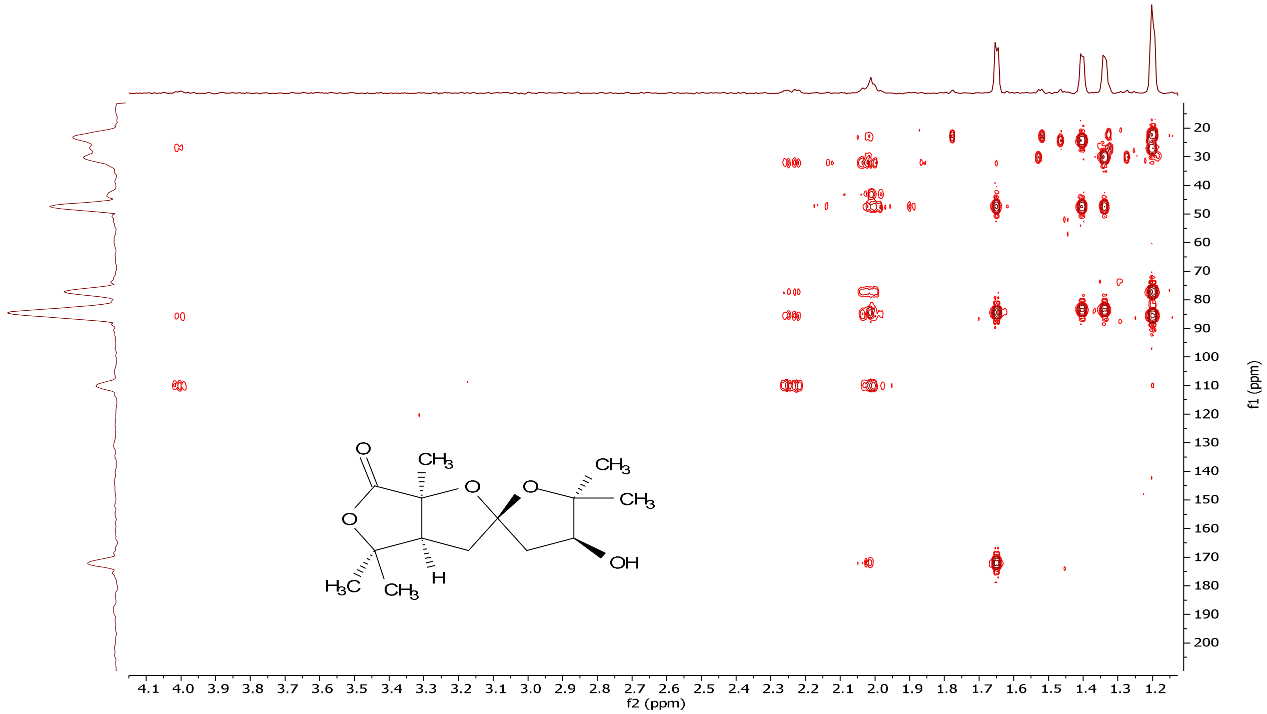

Figure S26. NOESY spectrum of compound 3 in CDCl₃.

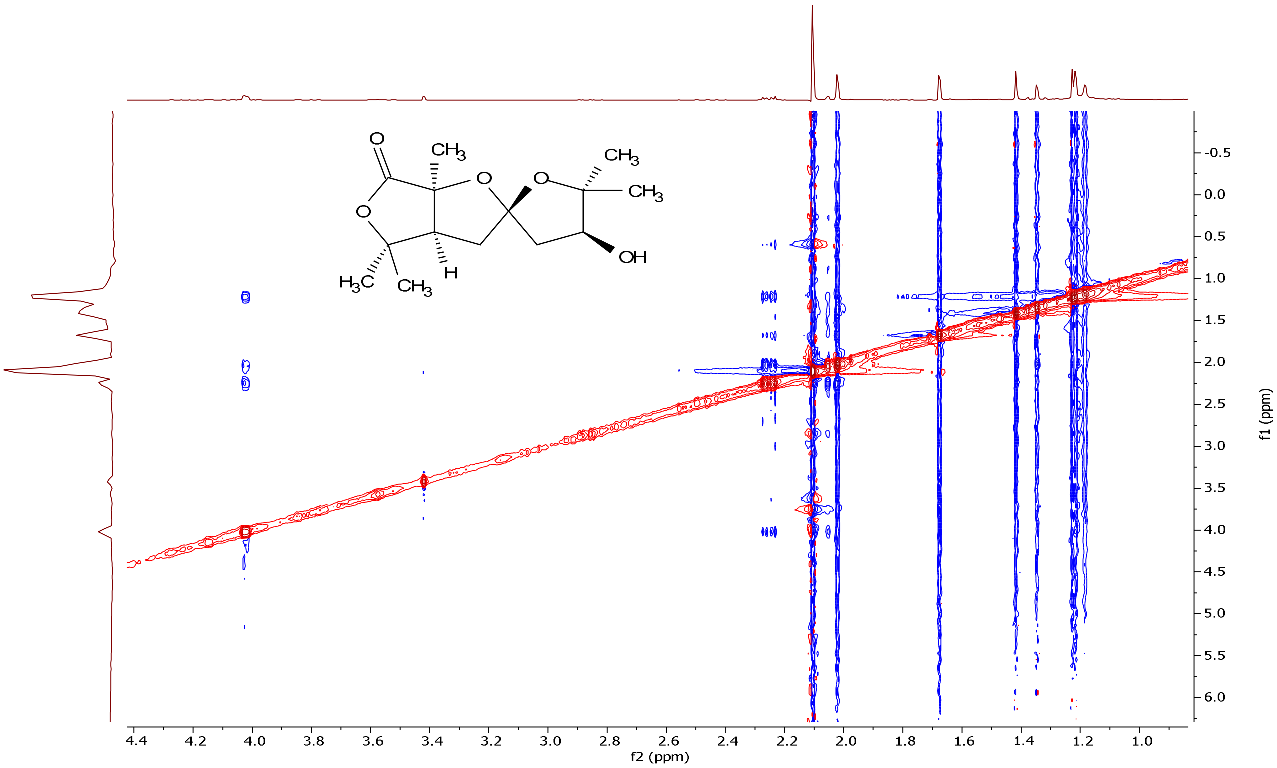

Figure S27. ^{1}H NMR spectrum (500 MHz) of compound 3 in C_5D_5N.

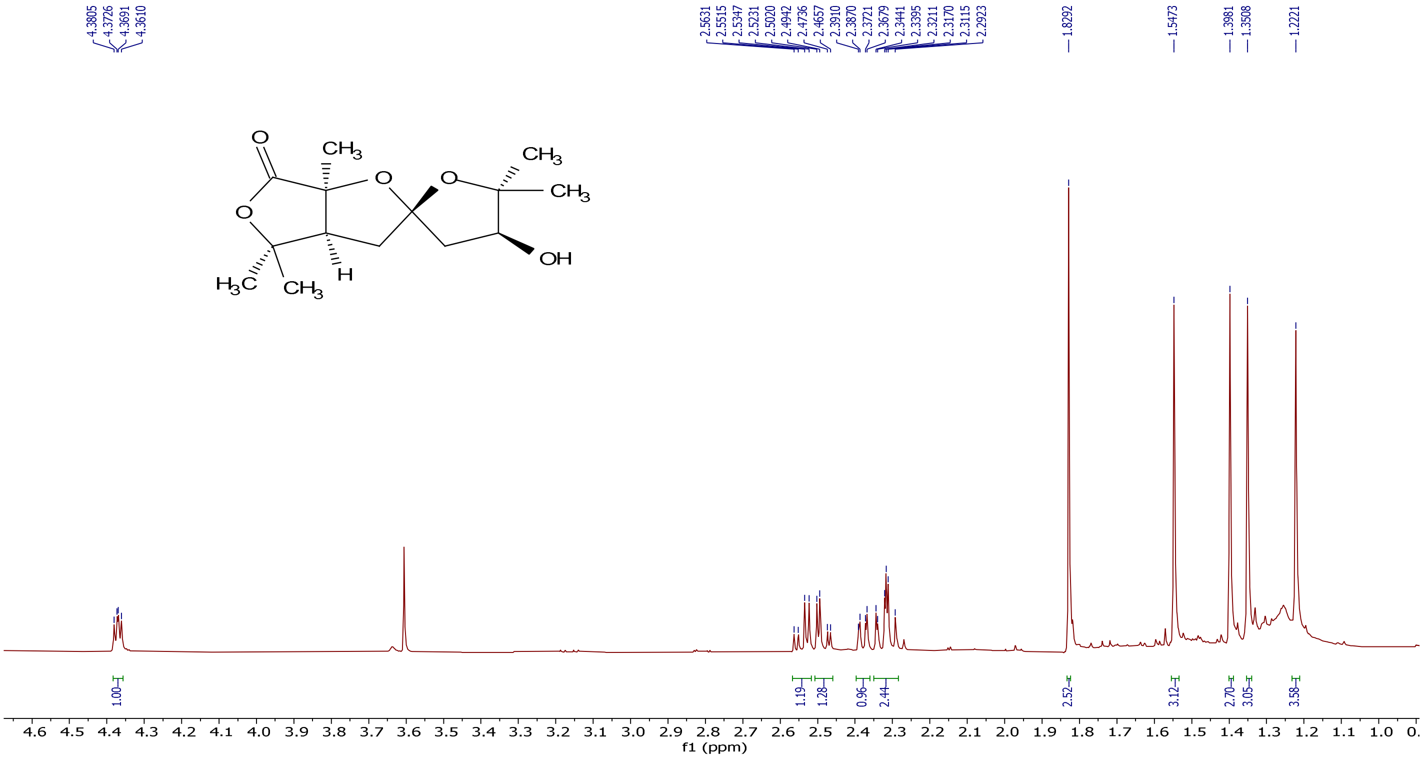

Figure S28. DEPT-Q spectrum (125 MHz) of compound 3 in C_5D_5N.

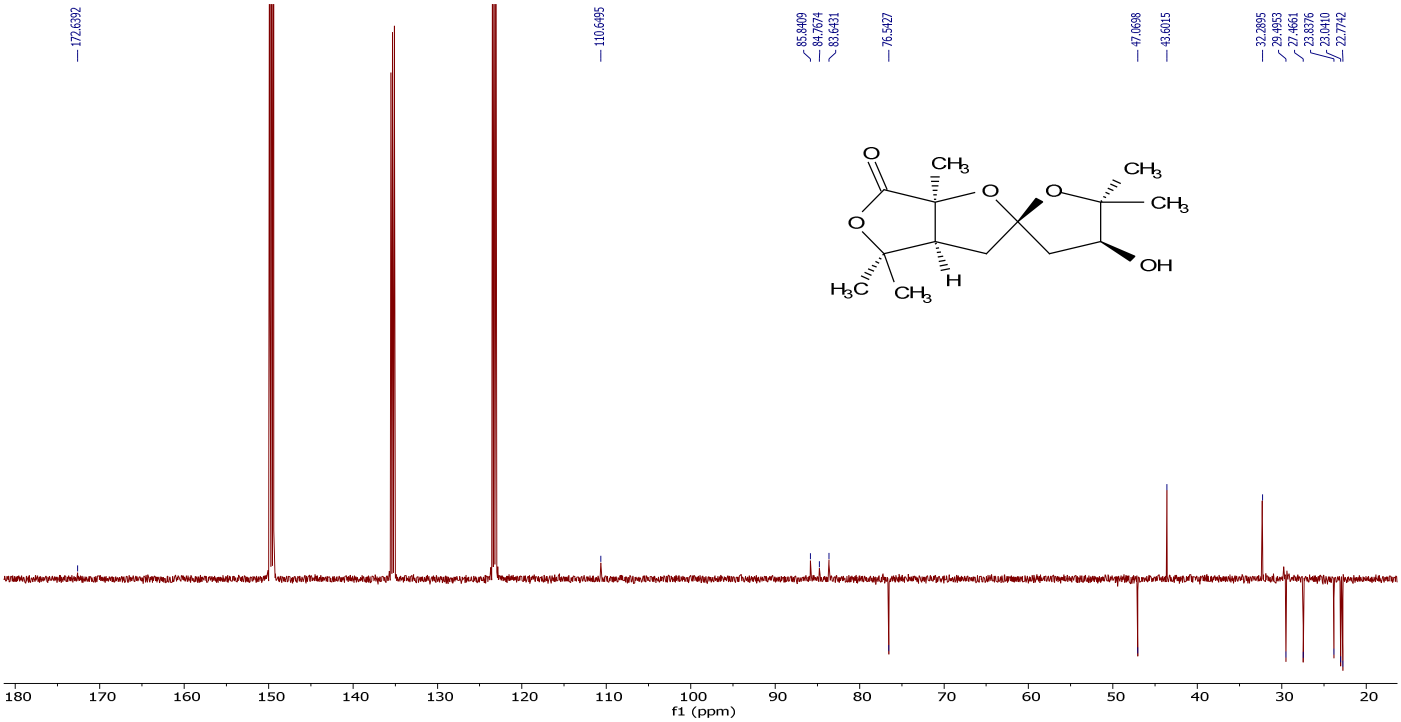

Figure S29. HSQC-DEPT spectrum of compound 3 in C_5D_5N.

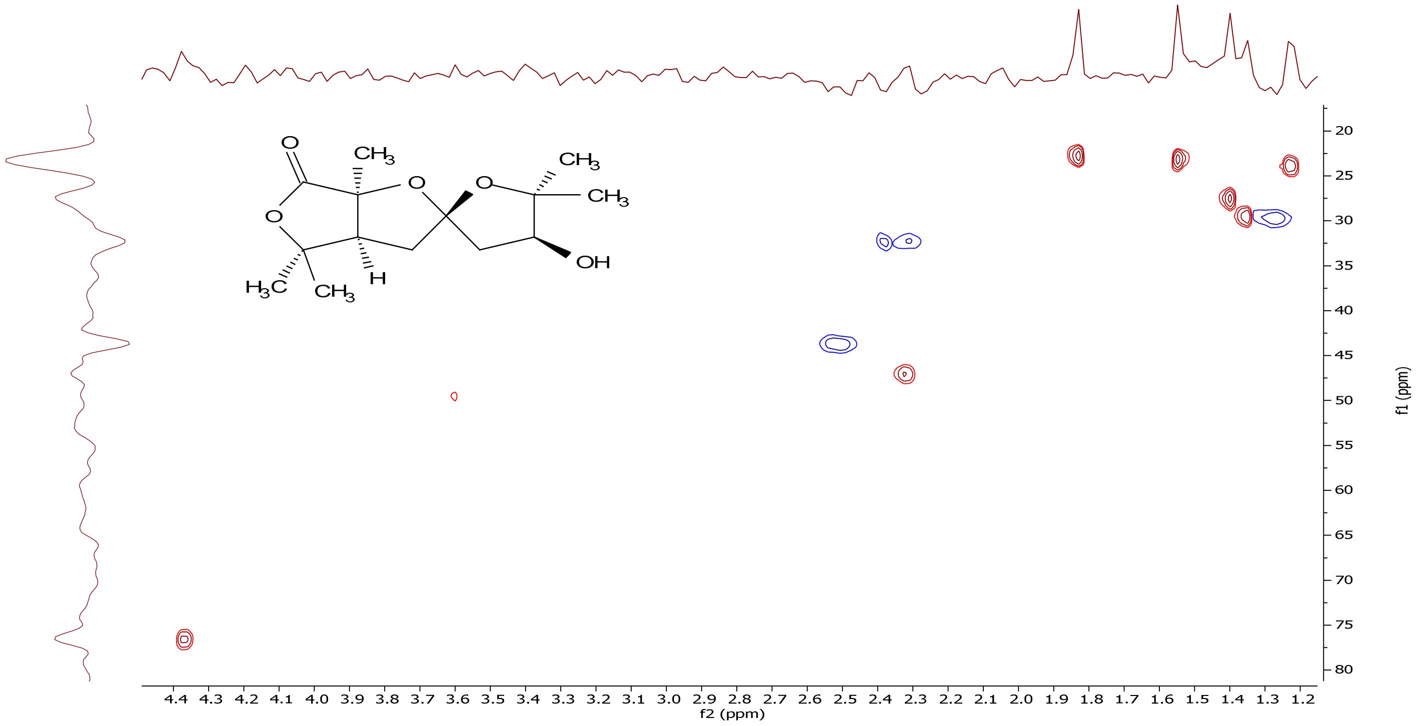

Figure S30. ^1H-^1H COSY spectrum of compound 3 in C_5D_5N.

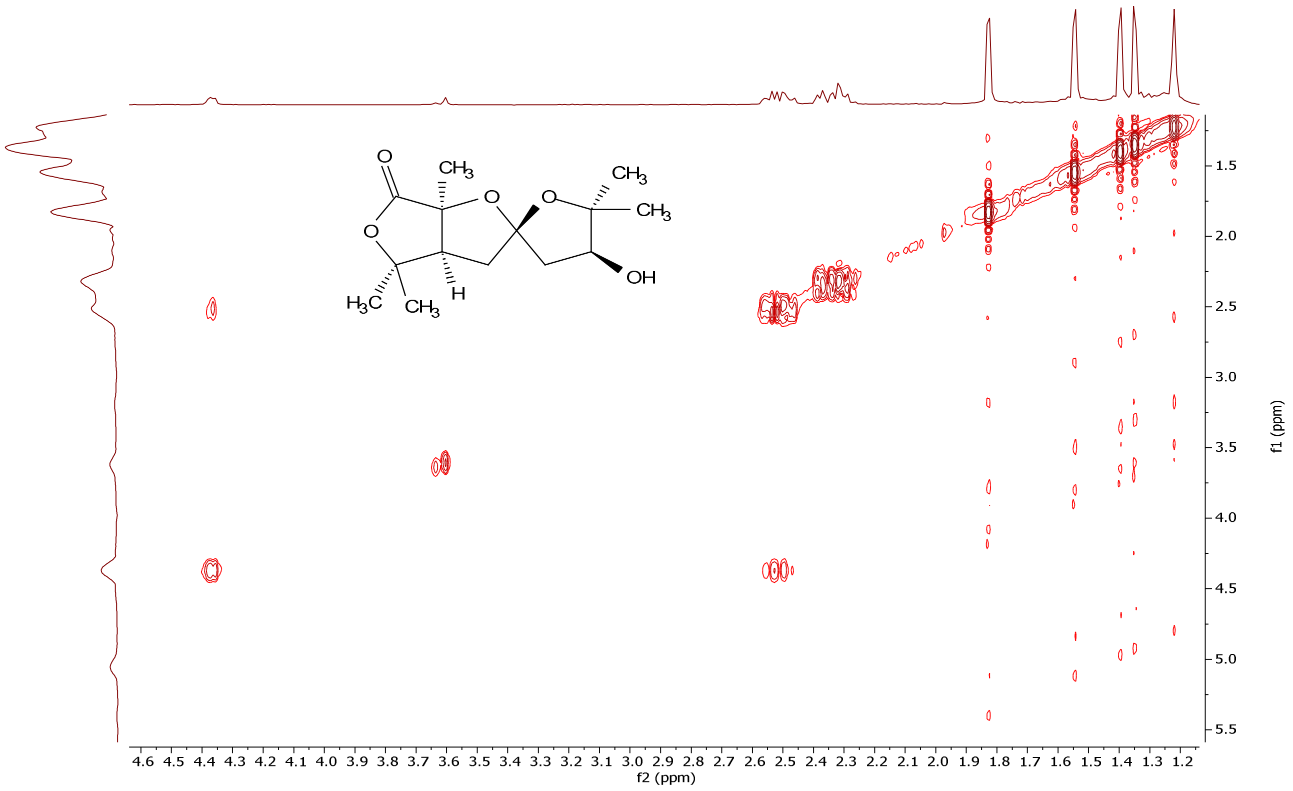

Figure S31. HMBC spectrum of compound 3 in C_5D_5N.

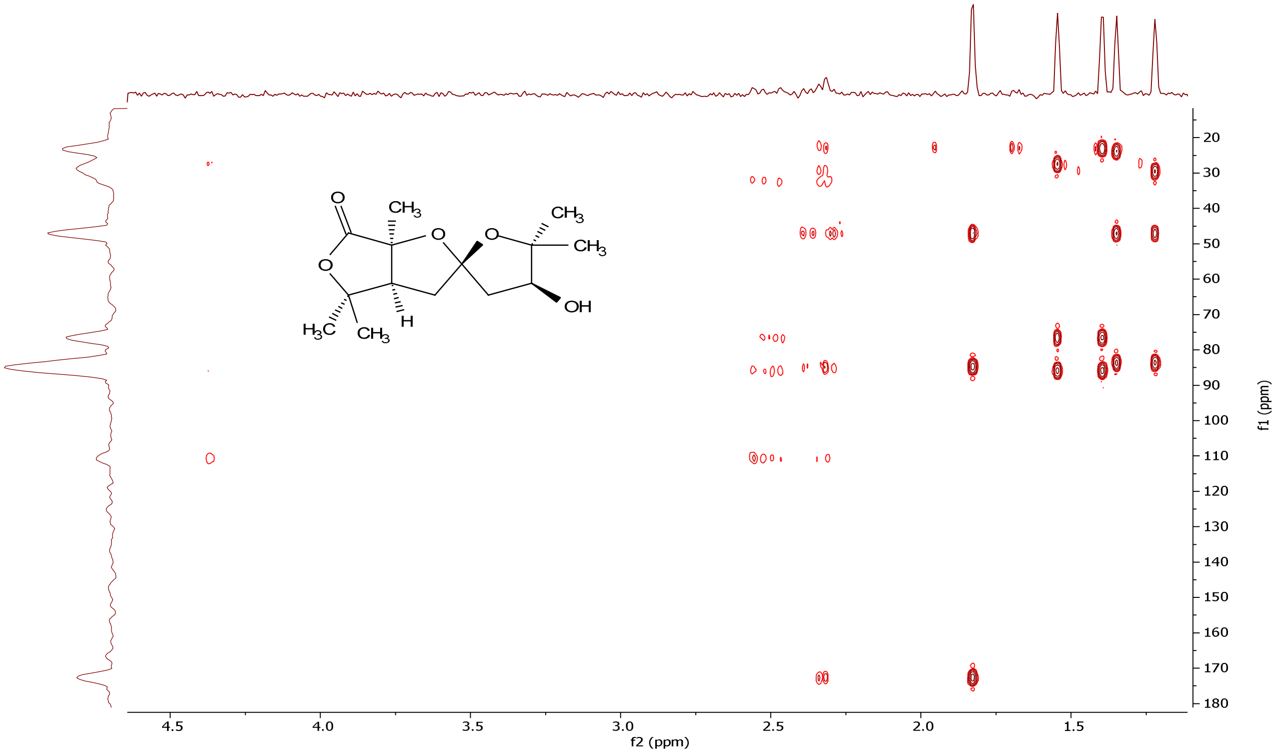

Figure S32. NOESY spectrum of compound 3 in C_5D_5N.

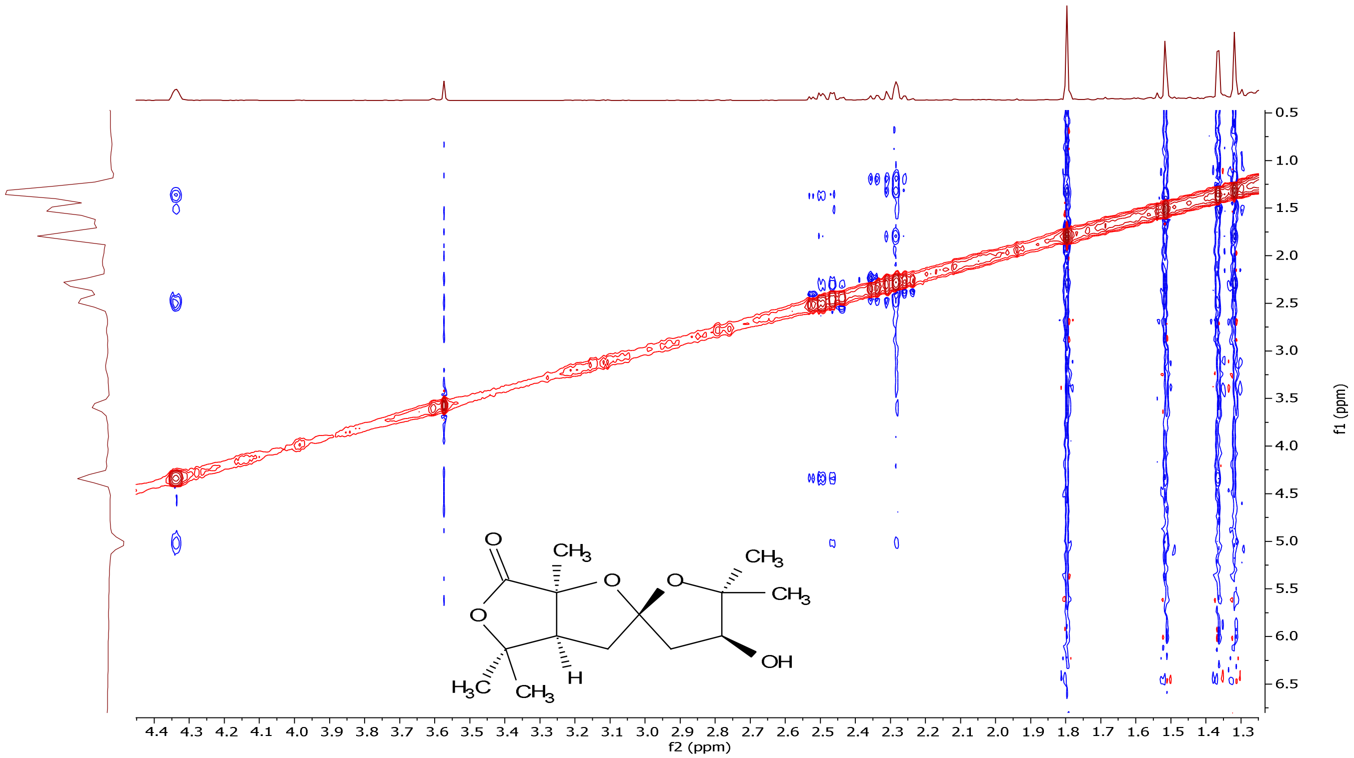

Figure S33. HRMS spectrum of compound 3.

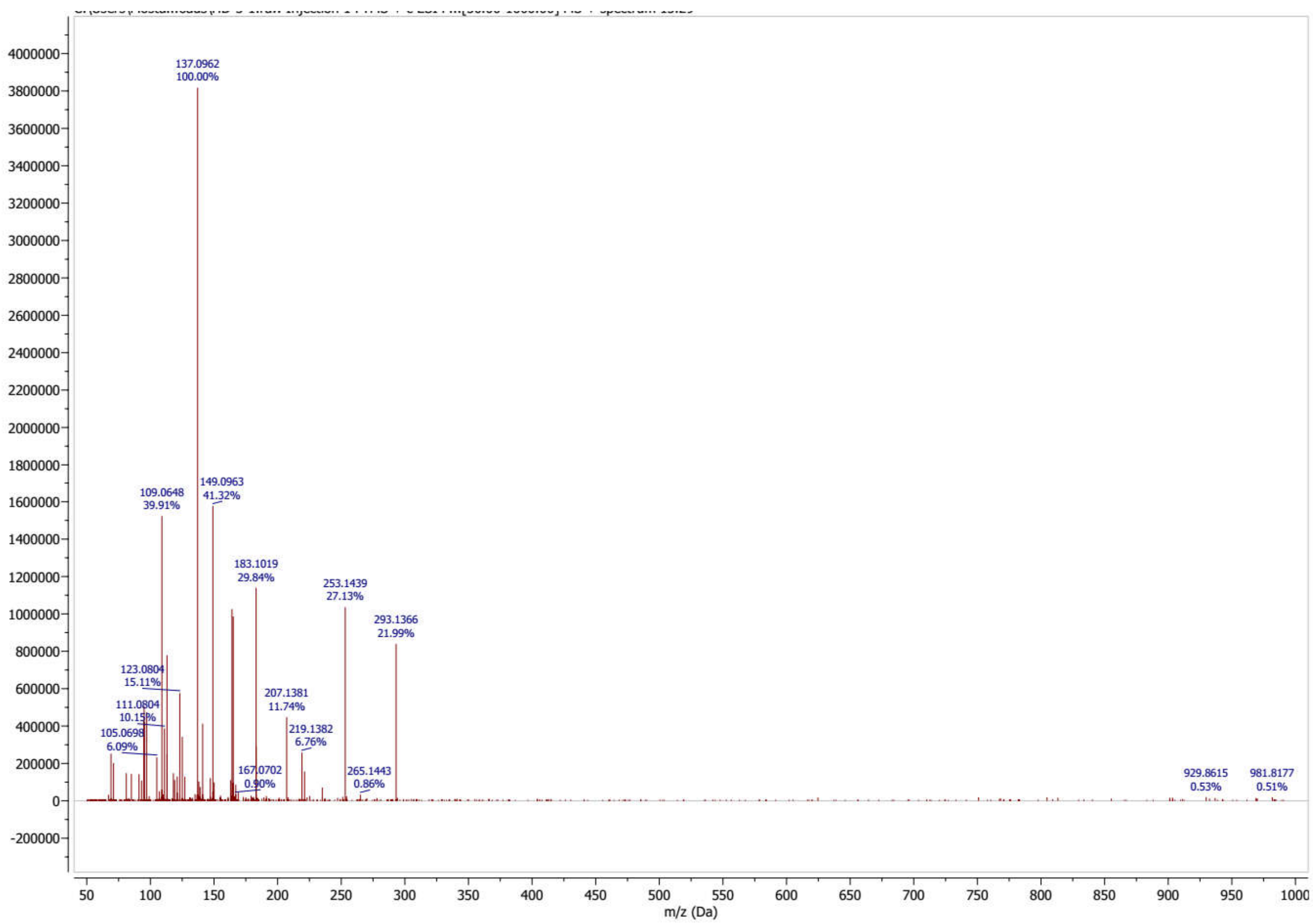

Table S4. Energies and Cartesian Coordinates (Å) for the Optimized Structure of compound 1

Electronic Energy = -1733.27842687

C	-0.66457	2.484193	-1.59343	C	0.322199	1.319776	-1.35593
C	-1.86649	2.124921	-0.6139	O	-1.88332	-2.02149	0.197359
O	-1.05231	-0.57815	-2.17084	C	-2.89144	-1.36545	0.057882
C	-0.76261	0.236525	-1.33345	C	-4.40883	-3.2973	0.240531
O	-1.13109	3.158827	1.487926	C	-5.67332	-3.86731	0.253742
O	-2.88057	-0.02225	-0.11144	C	-6.79811	-3.06359	0.088762
C	-2.42022	4.523104	-0.0479	C	-6.65804	-1.69035	-0.08949
C	-3.41384	2.667084	1.302863	C	-5.39442	-1.11521	-0.10335
C	-2.18275	3.125494	0.523898	C	-4.26601	-1.92033	0.062501
C	3.581568	-1.48785	3.306173	H	-1.02005	2.530248	-2.62373
C	5.310525	0.199899	2.561532	H	-0.1935	3.436027	-1.35182
C	3.849982	-0.14659	2.679829	H	-2.77231	2.052523	-1.22087
C	2.914641	0.705612	2.248324	H	-0.35916	3.601253	1.109994
C	1.422693	0.540193	2.300388	H	-2.68512	5.204539	0.763474
C	6.020292	-1.22878	-1.49532	H	-3.23635	4.516764	-0.77486
C	5.789013	-3.22433	0.044362	H	-1.53028	4.919887	-0.54347
C	5.098456	-2.22389	-0.84444	H	-3.23921	1.699279	1.774202
C	3.77381	-2.26235	-1.02076	H	-4.28443	2.586631	0.648137
C	2.940485	-1.36227	-1.89528	H	-3.63281	3.398001	2.084554
O	-1.30095	0.023103	2.148473	H	4.041778	-1.54414	4.298871
C	2.56573	2.039723	-2.36302	H	2.520793	-1.7146	3.41297
C	1.253866	0.329545	-3.53916	H	4.037504	-2.28369	2.706179
O	1.372178	2.671719	0.348507	H	5.793666	0.199176	3.545347
C	2.270186	-0.19228	-1.14933	H	5.839139	-0.54452	1.955092
C	1.371492	-0.73315	0.012992	H	5.460866	1.180994	2.106012
C	1.591403	0.872355	-2.13814	H	3.252136	1.643686	1.81335
C	0.878927	1.605894	0.04841	H	0.978696	1.413376	2.790636
C	0.758177	0.40794	0.925108	H	1.12878	-0.32794	2.892092
C	-0.78211	0.32424	1.103811	H	6.564977	-0.66013	-0.73315
C	-1.62963	0.628622	-0.16338	H	6.777409	-1.74347	-2.09783

H	5.503128	-0.51542	-2.13724	H	2.173014	0.208338	-4.11678
H	6.342062	-2.72438	0.847915	H	0.720422	-0.61527	-3.52495
H	5.081941	-3.92199	0.498946	H	3.079236	0.351007	-0.64583
H	6.526138	-3.80465	-0.52293	H	0.599796	-1.41217	-0.35397
H	3.226195	-3.03677	-0.4858	H	2.011304	-1.32998	0.663958
H	2.165282	-1.96718	-2.3782	H	-3.52027	-3.90477	0.367333
H	3.552678	-0.95207	-2.7019	H	-5.78359	-4.93727	0.392488
H	2.135452	2.769647	-3.05537	H	-7.7874	-3.50908	0.09902
H	2.830627	2.558542	-1.4425	H	-7.53571	-1.06632	-0.21792
H	3.486173	1.659989	-2.81628	H	-5.27904	-0.04724	-0.24127
H	0.632116	1.054428	-4.07286				

Table S5. Energies and Cartesian Coordinates (Å) for the Optimized Structures of compound 3

Conformer 1- Electronic Energy = -922.92529032

C	1.334348	-1.21674	-0.10924	H	0.327657	1.304755	-2.05816
C	1.7708	-0.10129	-1.09632	H	-1.97499	-1.63684	-1.99842
C	0.459117	0.247196	-1.8322	H	-2.3092	0.094535	-2.22518
C	-0.64435	-0.28315	-0.91673	H	-2.77475	-1.55768	0.301505
O	-0.06326	-1.45418	-0.3294	H	-4.44342	0.219882	-1.09713
C	-1.99608	-0.66624	-1.50068	H	1.903199	-2.9829	-1.20089
C	-2.8985	-0.62756	-0.26098	H	3.13066	-2.43623	-0.03712
C	-2.30131	0.548369	0.580745	H	1.661774	-3.24276	0.544215
O	-0.93531	0.687811	0.083496	H	4.455885	1.581544	0.391833
C	1.538864	-0.58872	1.281615	H	4.351273	-0.09184	-0.19078
O	2.148418	0.603188	1.187103	H	4.278445	1.252701	-1.34321
C	2.457184	0.986883	-0.20588	H	2.138107	2.768571	-1.38989
O	-4.2894	-0.53852	-0.51616	H	0.873831	2.469258	-0.17366
C	2.056674	-2.55412	-0.20719	H	2.467921	3.076449	0.322027
O	1.22576	-1.07595	2.346544	H	-2.48445	2.684785	0.82939
C	3.97991	0.926121	-0.34259	H	-3.0398	2.116318	-0.74888
C	1.945256	2.413404	-0.37336	H	-4.04126	1.848259	0.696191
C	-3.01521	1.87599	0.319374	H	-1.82285	1.054799	2.638967
C	-2.25442	0.223765	2.072405	H	-3.26579	0.040498	2.45035
H	2.506815	-0.47411	-1.8108	H	-1.64496	-0.66623	2.24766
H	0.422519	-0.30603	-2.77444				

Conformer 2- Electronic Energy = -922.92509492

C	1.334146	-1.21761	-0.10379	H	0.327518	1.297174	-2.06169
C	1.770184	-0.10648	-1.09601	H	-1.97452	-1.63921	-1.98964
C	0.457751	0.240511	-1.83091	H	-2.31829	0.092048	-2.21495
C	-0.64553	-0.28327	-0.91113	H	-2.80267	-1.55382	0.299243
O	-0.06407	-1.45328	-0.31907	H	-4.8225	-0.60479	0.09306
C	-1.9964	-0.6672	-1.49454	H	1.659741	-3.24214	0.555402
C	-2.90578	-0.61989	-0.26846	H	1.897563	-2.98833	-1.191
C	-2.30552	0.552524	0.578878	H	3.128772	-2.44019	-0.03188
O	-0.93635	0.690643	0.082947	H	4.278028	1.245318	-1.3539
C	1.543863	-0.58525	1.284284	H	4.460087	1.57987	0.379572
O	2.154041	0.606061	1.183867	H	4.352834	-0.09538	-0.19718
C	2.459397	0.984434	-0.21106	H	2.138854	2.762602	-1.40024
O	-4.25508	-0.43957	-0.67218	H	0.876781	2.467497	-0.18068
C	2.054157	-2.55656	-0.19907	H	2.472067	3.075703	0.310074
O	1.234083	-1.06845	2.352017	H	-4.0354	1.86482	0.709913
C	3.981772	0.922334	-0.35145	H	-2.46874	2.688791	0.818924
C	1.947812	2.410683	-0.38225	H	-3.06175	2.103452	-0.74899
C	-3.01419	1.881918	0.320371	H	-3.25498	0.058824	2.467135
C	-2.24724	0.223205	2.069952	H	-1.65135	-0.67751	2.237212
H	2.504689	-0.48303	-1.81011	H	-1.79298	1.045983	2.630683
H	0.418619	-0.31698	-2.77055				

Conformer 3- Electronic Energy = -922.92451963

C	1.342985	-1.2225	-0.08174	O	-4.27412	-0.43409	-0.49488
C	1.772161	-0.12519	-1.09238	C	2.070515	-2.55875	-0.15505
C	0.456707	0.202239	-1.83118	O	1.240948	-1.03246	2.371284
C	-0.64244	-0.31211	-0.90079	C	3.979073	0.927635	-0.36732
O	-0.05388	-1.46973	-0.29247	C	1.937049	2.404037	-0.41877
C	-1.99576	-0.71021	-1.4714	C	-3.00591	1.896879	0.197209
C	-2.89335	-0.63248	-0.22988	C	-2.29966	0.345098	2.061451
C	-2.31298	0.579694	0.551949	H	2.507913	-0.50947	-1.80106
O	-0.93222	0.675754	0.08259	H	0.419541	-0.37152	-2.76104
C	1.549308	-0.56596	1.295744	H	0.320421	1.254167	-2.0795
O	2.155056	0.625743	1.175428	H	-1.97131	-1.69533	-1.94115
C	2.456578	0.983418	-0.22584	H	-2.32238	0.029444	-2.20934

H	-2.74953	-1.53942	0.372119	H	0.866115	2.458126	-0.21621
H	-4.62916	-1.26859	-0.82921	H	2.458834	3.0827	0.262098
H	1.679789	-3.23417	0.610422	H	-2.46519	2.726378	0.662492
H	1.916578	-3.00758	-1.13983	H	-3.02291	2.05919	-0.88374
H	3.144429	-2.43377	0.010342	H	-4.03603	1.903331	0.560881
H	4.272171	1.23678	-1.37507	H	-1.87556	1.207466	2.585078
H	4.454518	1.599149	0.352785	H	-3.32027	0.190901	2.426928
H	4.356197	-0.0854	-0.19789	H	-1.7006	-0.53581	2.305685
H	2.124533	2.740923	-1.44252				

Conformer 4- Electronic Energy = -922.91870672

C	1.248268	0.853962	0.44621
C	1.581848	-0.64531	0.638323
C	0.399316	-1.33731	-0.05852
C	-0.7627	-0.35744	0.092895
O	-0.12935	0.95041	0.06615
C	-1.65705	-0.47476	1.328928
C	-2.91528	0.264323	0.883411
C	-3.00573	-0.08845	-0.63774
O	-1.63732	-0.45374	-1.00199
C	2.132954	1.242263	-0.75506
O	3.053775	0.285782	-0.99496
C	2.980821	-0.82159	-0.01718
O	-4.02634	-0.1483	1.664574
C	1.496202	1.796515	1.623431
O	2.055801	2.249517	-1.42176
C	4.120482	-0.62812	0.986091
C	3.187505	-2.11237	-0.80425
C	-3.89006	-1.30574	-0.90597
C	-3.41752	1.109306	-1.49271
H	1.625649	-0.92395	1.694001
H	0.173193	-2.32414	0.348832
	0.575231	-1.44467	-1.13233
H	-1.2002	-0.05716	2.227839
H	-1.90418	-1.52539	1.515774
H	-2.76041	1.346531	0.985258
H	-4.75678	0.4619	1.494475
H	0.889642	1.487277	2.478537
H	2.545651	1.81113	1.928092
H	1.210476	2.812029	1.336481
H	5.079851	-0.60011	0.462613
H	4.013735	0.297277	1.556104
H	4.137258	-1.46271	1.692977
H	3.070009	-2.97539	-0.14253
H	2.478213	-2.20687	-1.6282
H	4.198292	-2.13696	-1.22059
H	-4.93855	-1.08181	-0.69315
H	-3.80445	-1.5951	-1.95764
H	-3.59167	-2.15609	-0.2873
H	-3.4127	0.848282	-2.55536
H	-4.42776	1.441206	-1.22987
H	-2.7274	1.943525	-1.33725
